# A Sensory-Motor Control Model of Animal Flight Explains Why Bats Fly Differently in Light Versus Dark

**DOI:** 10.1371/journal.pbio.1002046

**Published:** 2015-01-28

**Authors:** Nadav S. Bar, Sigurd Skogestad, Jose M. Marçal, Nachum Ulanovsky, Yossi Yovel

**Affiliations:** 1 Department of Chemical Engineering, Norwegian University of Science and Technology (NTNU), Trondheim, Norway; 2 Institute for Telecommunications, University of Lisbon, Lisbon, Portugal; 3 Department of Neurobiology, Weizmann Institute of Science, Rehovot, Israel; 4 Department of Zoology, Faculty of Life Sciences, and Sagol School of Neuroscience, Tel Aviv University, Tel Aviv, Israel; Lund University, SWEDEN

## Abstract

Animal flight requires fine motor control. However, it is unknown how flying animals rapidly transform noisy sensory information into adequate motor commands. Here we developed a sensorimotor control model that explains vertebrate flight guidance with high fidelity. This simple model accurately reconstructed complex trajectories of bats flying in the dark. The model implies that in order to apply appropriate motor commands, bats have to estimate not only the angle-to-target, as was previously assumed, but also the angular velocity (“proportional-derivative” controller). Next, we conducted experiments in which bats flew in light conditions. When using vision, bats altered their movements, reducing the flight curvature. This change was explained by the model via reduction in sensory noise under vision versus pure echolocation. These results imply a surprising link between sensory noise and movement dynamics. We propose that this sensory-motor link is fundamental to motion control in rapidly moving animals under different sensory conditions, on land, sea, or air.

## Author Summary

Bats are extremely skillful aviators: they are able to capture prey and land on targets under challenging flight conditions, as well as maneuver accurately using either echolocation or vision. It remains a mystery, however, how bats—or other flying animals—rapidly translate the noisy incoming sensory information into correct motor commands in order to converge onto a target. To address this question, we developed a sensorimotor control model that explains animal flight guidance and tested it in bats with experiments conducted under dark and light conditions. The model reproduced the bats’ flight trajectory with very high accuracy, suggesting that bats have to estimate not only the angle to target but also changes in the angle over time (angular velocity). Additionally, we demonstrate that the bat must suppress its sensory noise by integrating sensory information over several sonar pulses in order to successfully guide its flight. Comparisons of flight trajectories in light and dark suggest that the surprisingly curved flights exhibited by bats in the dark are due to sensory noise, not motor limitations. We hypothesize that rapidly moving animals must adaptively change their motor control strategy to optimally match the sensory conditions.

## Abstract 3

A model of animal flight guidance suggests that bats use estimates of angular velocity and time-integrated sensory information to find their targets, and explains why bats fly straighter in the light than in the dark.

## Introduction

**Figure 1:**
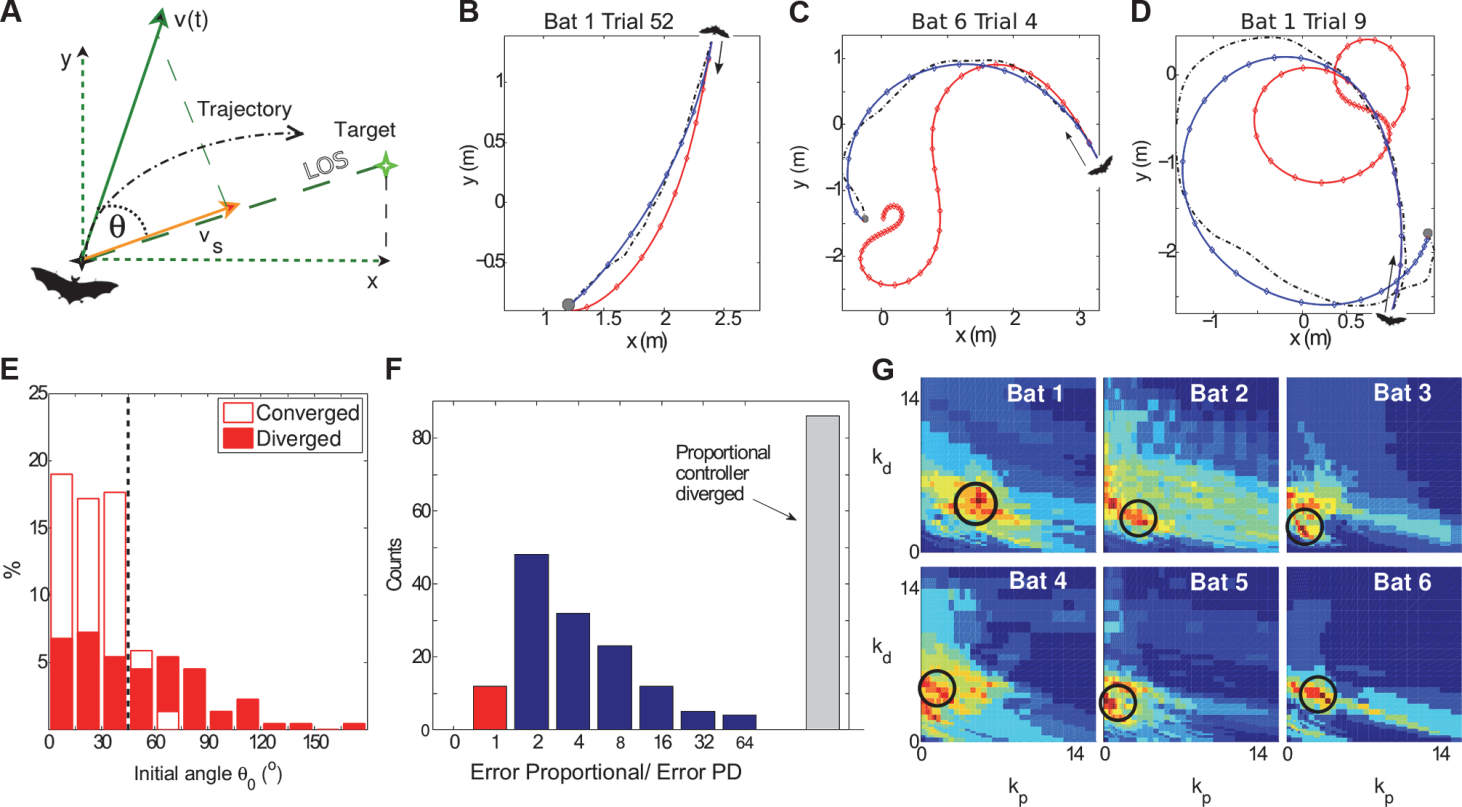
Control theory sensorimotor models for bat flight behavior. (a) Schematic top-view depiction of a bat trajectory (black dashed line), together with the line of sight (LOS) to the target, the velocity *ν*(*t*), and the angle *θ*(*t*) between *ν*(*t*) and the LOS. (b–d) 2-D projections of actual bat flight trajectories (black dashed curves) together with the simulated trajectories from the proportional controller (red) and the proportional-derivative (PD) controller (blue). Examples b–d are ordered according to the curvature of the trajectory. For small initial angles *θ*_0_ (illustrated by example b, *θ*_0_ = 18°), convergence is achieved by both controllers, but is more accurate with the PD controller (blue). Higher initial angles (c, *θ*_0_ = 77°) require a controller that considers the angular velocity in addition to the angle *θ*, and such PD controllers achieved very good performance (blue curves in example c). Example d shows a trial with a small initial angle (*θ*_0_ = 9°) that did not converge, probably because the target was too close and the bat did not manage to slow down on time. Dots (blue or red) represent sensory acquisition time samples (10 Hz echolocation, i.e., a dot every 100 ms). Gray sphere depicts the 10-cm diameter landing target, drawn to scale. (e) Histogram of the initial angle *θ*_0_ of the converging (open bars) and diverging trials (full bars), based on a proportional controller. The proportional controller was able to achieve convergence mostly for small angles (below 45°—vertical black dashed line). (f) Histogram of the error ratio (error in proportional controller / error in PD controller). Grey bar, number of simulations that completely diverged when using the Proportional controller. Blue or red bars, number of trials in which the PD controller was more accurate than the proportional controller (ratio >1, blue), or vice versa (ratio <1, red). PD control is clearly superior to proportional control. (g) 2-D histograms of the parameter space (*k_d_* and *k_p_* ) that best fit the real trajectories, plotted individually for each of the 6 bats; based on all the 222 trials in the dark condition. Colors represent the number of trials for which a certain *k_p_, k_d_*, combination was found to best reconstruct the real trajectory (see Materials and Methods). Color scale, from zero (blue) to maximum count (red). Red represents the following values in bats #1–6, respectively: 13, 19, 10, 12, 14, 7. A clear optimum is visible for each bat. Circles represent the optimum values (see Materials and Methods), which are fixed for the simulations of each bat. All the data is available in the S1 Data.

An important open question in neuroscience is how animals transform incoming sensory information into motor commands to control movement [1–6]. This problem is particularly difficult in flying animals, in which sensory input is rapid and motor control must be accurate. Bats are the only mammals capable of true flight [7,8]. Many tasks performed by bats require fine maneuvering toward targets (e.g., catching mobile prey or landing on small stationary objects). To converge to its target, the bat must reduce to zero the azimuth-to-target angle, *θ* (Fig. 1A). Reducing *θ* requires careful, well-coordinated control of the forces the bat applies to maneuver. Thus, a major challenge the bat faces is to translate the noisy estimates of *θ* into motor commands.

A few previous studies tested how bats control their approach [9,10]. These studies focused either on the flight pattern [10–13] or on the echolocation dynamics [9,10,14], but they did not examine the sensorimotor loop [15]—that is, how bats rely on sensory input to control flight motor commands. Here, we aimed to examine: (1) If it is enough for the bat to measure the azimuth-to-target (*θ*), or does it need additional information in order to apply the correct motor commands to converge onto the target? Specifically, it is well known from control theory that noisy measurements can pose a great challenge in fine guidance tasks, necessitating additional measurements, such as derivatives of *θ*. (2) Does the bat change its control strategy under different sensory conditions which differ in their noise level, such as in dark versus light, and moreover, do environments with reduced sensory noise affect the motor commands and accordingly the flight? To answer these questions, we developed a control-theory based sensorimotor model that receives noisy sensory input and computes the needed forces based on basic physical principles. This model allowed us to test different control strategies to evaluate the bat’s sensorimotor strategy and to examine its control strategy under different sensory conditions (light versus dark). To test our model, we used behavioral data from Egyptian fruit bats (Rousettus aegyptiacus)—flying mammals that possess an advanced biosonar (echolocation) system [16–21], as well as an excellent visual system [22].

We found that a simple model, which considers only the angle-to-target and its derivative (a “proportional-derivative controller”), was able to reconstruct complex, several-meter-long flight trajectories very accurately—with an average error of only 14.6 cm. We show that, to successfully land in the dark, the bat has to integrate (to average) sensory data from its biosonar system over several hundred milliseconds, in order to overcome sensory noise. We also demonstrate that when flying in light and using vision, bats reduced their flight curvature. This change in movement pattern was fully explained by the model as resulting from the reduced sensory noise when using vision. Taken together, these results imply an important link between sensory noise and movement dynamics in a rapidly moving mammal.

## Results

Bats were trained to land on a 10 cm diameter sphere placed at a random position in a 6 × 7 m large room. We started off with experiments in complete darkness, in which the bats had to rely exclusively on biosonar to estimate the angle-to-target *θ* using acoustic cues extracted from the reflected echoes [14,17,22,23]. In complete darkness, Egyptian fruit bats emit biosonar pulse-pairs at a rate of 7–10 Hz [17,20], and hence in the model we assumed a 10 Hz sensory acquisition rate. Interestingly, bats often did not fly to the target in a direct line, but rather exhibited a curved trajectory, sometimes even circling around the target (Fig. 1B-D, black dashed curves; a total of 222 trials were analyzed). We aimed to elucidate this behavior. Our model received as input only the initial conditions of the real bat: initial flight angle *θ*_0_, initial velocity *ν*_0_, the initial position of the bat and the target, as well as a fixed controller parameter determined from a training set that contained 80% of all the trials for each bat (see details in the text below and in the Materials and Methods). The model then simulated the full flight trajectory according to a sensorimotor control model and Newton’s laws of motion, until landing on the target (or missing it). The simulated bat began its flight at the real bat’s origin and with the same direction and speed as the real bat. From this instant onwards, at every sensory update (see below) the simulated bat re-estimated the direction and distance of the target (relative to the bat’s new position) and updated its next motor commands accordingly. In our model, the distance to the target was only used to slow down the bat as it approached landing and not to guide it. We assumed that distance is estimated via bio-sonar. The main objective of the sensorimotor control model was to translate the sensory azimuth measurements (direction to target) into motor commands. We tested several paradigms to perform this translation, comparing them to the bats’ behavior (see below).

### A Proportional Controller Fails to Explain Bat Flight Behavior

We simulated several control strategies with increasing complexity; in all models, turning forces *F*_⊥_ were applied perpendicularly to the movement trajectory in order to turn (maneuver) the bat based on the sensory input.

We first tested the simplest possible control strategy, the so-called “proportional control” [24–26], in which the turning forces *F*_⊥_ at every moment are proportional to the measured angle *θ*:

$$F_{⊥}=-k_{p}\cdot \theta$$

where *k_p_* is a constant, referred to as the “proportional gain” (Materials and Methods). Such a simple proportional controller performed poorly: First, it converged onto the target in only 61% of the complete-darkness trials (136/222). Second, it converged successfully only in trials with “easy” initial conditions, i.e., small initial angle *θ*_0_; 88% of the successful trials (120/136) had *θ*_0_ < 45° (see, for example, red curve in Fig. 1B). The convergence in these trials is not surprising because the bat started flying already heading towards the target, and thus small corrections were sufficient to reach the target. Third, in the difficult cases with *θ*_0_ > 45°, the model failed in 73% of the trials (*θ*_0_ > 45° occurred in 59/222 trials, and in 43/59 or 73% of these difficult trials, the model failed to converge; Fig. 1E, compare empty versus full bars to the right of the dashed line [*θ*_0_ > 45°]; c.f. Fig. 1C). Failures to converge occurred mostly because the forces applied during flight were too large, leading to large angular velocity and oscillations of *θ* that did not converge to zero (see S1 Fig., middle row). Thus, a simple proportional controller is not adequate to explain bat guidance of flight.

### A Proportional-Derivative Controller Accurately Reconstructs Bat Flight Trajectories

The failure of the proportional controller implies a need for a stabilizing effect, to counteract these strong forces. A simple stabilizer commonly used in control theory is to add a derivative term, which dampens the overshooting effect of the proportional controller; such a control strategy is called a “proportional-derivative (PD) controller” [24–27]:

$$F_{⊥}=-k_{p}\cdot \theta -k_{d}\cdot d\theta /dt$$

The PD controller proved to be dramatically superior as compared to the simpler proportional controller: the PD controller led to convergence in 100% of the trials (222/222, see examples in Fig. 1B-D, blue lines); it converged successfully even in those trials in which the bat maneuvered strongly (Fig. 1C,D: compare the red lines [proportional controller] that diverged versus the blue lines [PD controller] that converged accurately; see more examples in S2–S7 Figs.). Notably, the PD controller was able to explain even extreme-maneuvering trials in which the bat exhibited a full 360° rotation (Fig. 1D). For each trial we found the model parameters (*k_p_* and *k_d_* ) that minimized the error between the simulated trajectory and real bat trajectory (see Materials and Methods for error-index definition). We computed the error index for the proportional controller and for the PD controller, for those 136 trials in which the proportional controller converged. The analysis revealed that in 92% of the cases (125/136), the PD controller led to trajectories with a smaller error (Fig. 1F, blue bars). The average reconstruction error of the PD controller over the testing data was extremely small—only 14.6 ± 1.1 cm (mean ± S.E.M.; see Materials and Methods for details on the training and testing procedure; see also S1 Table for data on each individual bat). In contrast, the proportional controller did not converge at all in 39% of the trials (86/222; Fig. 1F, gray bar), and in those trials where it did converge, its trajectory error was, on average, 3.6 times worse than in the PD controller. In some cases (12.5%, 17/136), the PD controller outperformed the proportional controller by more than 10-fold.

Importantly, when we plotted the distribution of the parameters (*k_p_*, *k_d_*) that best fitted each experiment, we found that bats used one consistent control strategy (Fig. 1G, note the uni-modal distribution of best parameters for each individual bat, peaking at around *k_d_* ≈ 2–6 and *k_p_* ≈ 1–5). When examining different individual bats, each bat had slightly different optimal parameters (Fig. 1G and S2 Table), but they were all centered within the same limited range in the parameter space. This consistency between and within bats, all of which exhibit high (non-zero) values of optimal *k_d_*, argues against the proportional model—and emphasizes the need for differentiating the angle, as implemented in the PD model.

Finally, we tested two alternative two-parameter models, the proportional-integrative (PI) model and the derivative-integrative (DI) model. Both of these models performed worse than the PD model, and one of them performed worse than the Proportional model as well (S8 Fig). We therefore conclude that the superior performance of the PD controller was specifically due to the combination of the proportional and derivative terms (P+D). This combination is particularly effective during rapid maneuvering because the proportional term corrects the error and the derivative term stabilizes the controller.

### Integrating Sensory Information Over Time Stabilizes Motor Performance

**Figure 2:**
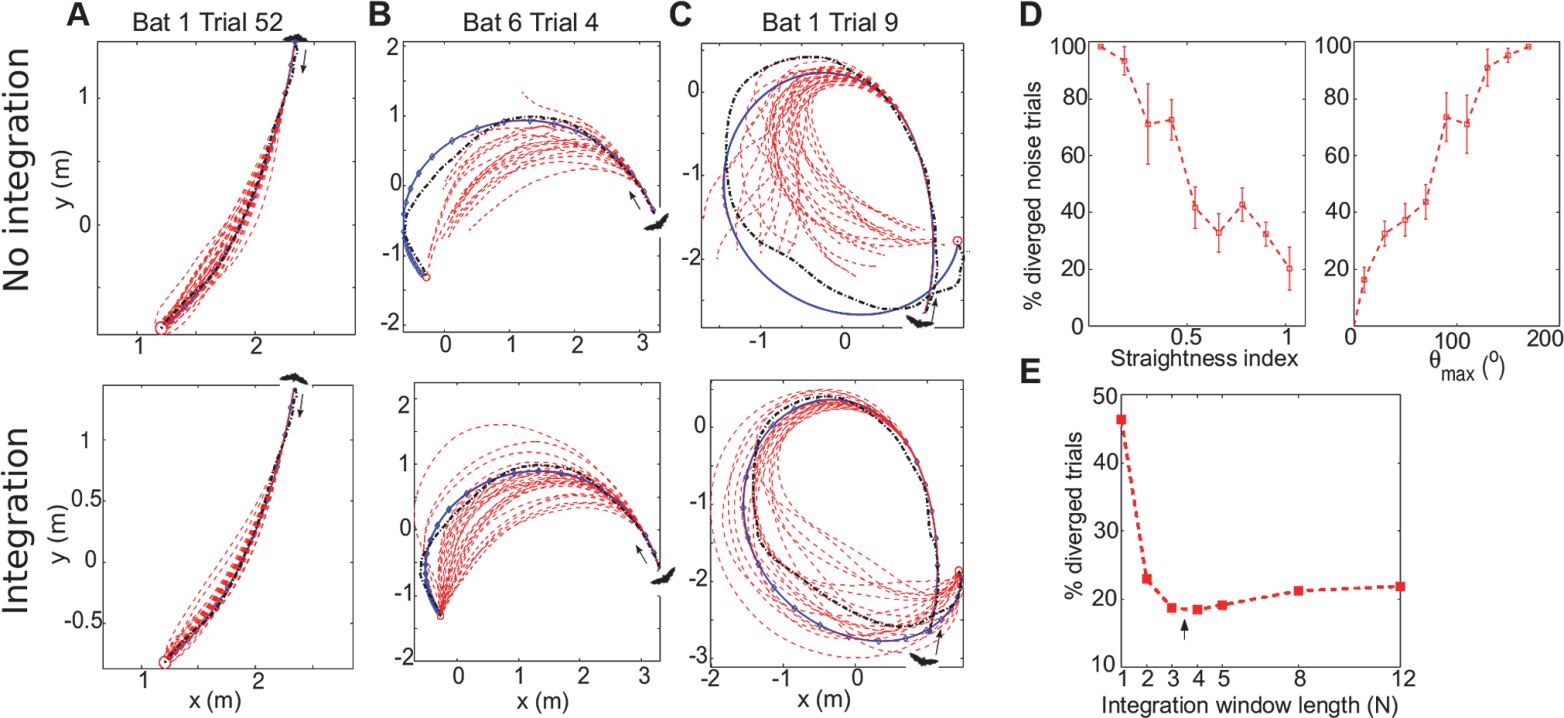
The effect of noise on sensorimotor control. (a–c) The same three example flight trajectories (trials) as in Fig. 1B–D. For each trial we performed 150 noise simulations (30 shown: red dashed lines), with additive Gaussian noise. Top row, without noise filtering; Bottom row, with an exponentially decaying weighted noise-suppression strategy. Black dashed curve, original trajectory; blue curve, PD simulated trajectory with no noise (deterministic); red curves, 30 simulated trajectories with noise (terminated after 7 s of simulated flight because bats never flew more than 5 s). (d) Percentage of diverged flights (cases with noise in which the simulated bat failed to converge to the target), plotted as a function of the straightness index (left) and the maximal angle *θ*_max_ (right). Error bars, mean ± S.E.M., computed over trials. Note that the percentage of diverged flights (failures) increase for more curved flights. (e) An integration (averaging) window of length 3–4 yielded simulations that reconstructed most accurately the real flight trajectories in the dark (arrow). The data is available in the S2 Data.

All the models we discussed so far were deterministic and assumed that the bat can perfectly measure the angle-to-target. In reality, however, all organisms have sensory errors. To assess the effect of this sensory noise, we tested two models of angle-dependent additive Gaussian noise, which mimic the sensory errors found in the auditory system of several vertebrates (S9 Fig. and Materials and Methods) [28–30]. As expected, sensory noise had strong implications for the convergence of the model: in many trials, adding the noise resulted in increased maneuvering errors (Fig. 2A, top, red dashed curves), and oftentimes led to complete failure to converge (Fig. 2B-C, top). The noise impaired convergence in 85% of the 222 trials (189/222). The percent of diverging simulations increased in trials with highly curved turns, as expressed by their small straightness index and large maximal angle *θ*_max_ (Fig. 2D, see Materials and Methods for definitions of these indices). Even in the trials that did converge despite the noise, the error index increased substantially, on average by more than 2-fold.

The model failed to converge in the presence of noise because the derivative term of the PD controller differentiates noisy measurements, which further amplifies the noise (see Materials and Methods). We therefore hypothesized that the bat must use a noise-suppression strategy and integrate (average) over several sensory measurements to overcome the noise. To elucidate the number of measurements of *θ* that should be averaged before executing the motor commands, we tested several different integration functions and evaluated their effect on the percent of successful convergences and on the error index. An exponentially decaying integrator (Materials and Methods), which only takes into account the three to four most recent measurements, was found to outperform uniform and linearly decaying integrators (S10 Fig.). This simple exponential integrator exhibited successful noise suppression and reproduced the bat’s flight trajectories with high fidelity (Fig. 2E, arrow; see also Fig. 2A-C, bottom—compare to the failed convergences in the top panels when not using noise suppression). Interestingly, there is a biologically-plausible update algorithm to implement such an exponential integrator, by only requiring the bat to remember the last *average* *θ* (see Materials and Methods). Previous work has shown that in stationary micro-bats (*Eptesicus fuscus*) that perform a detection task, integrating information over five to ten sonar calls substantially improves performance [31]. Our current findings strengthen the need for integration. The shorter integration window that we found in flying bats (three to four sonar pulses; note the optimum in Fig. 2E: arrow) versus stationary bats (five to ten calls [31]) might reflect the need for faster decision-making during flight maneuvers. It might also result from the difference between the bat species used in the two studies, which rely on echolocation to a different extent (*Rousettus* less than *Eptesicus*).

### Experiments in Light Versus Dark Reveal That Sensory Noise Affects the Motor Strategy

An important prediction of our model is that sensory noise determines motor performance. In particular, the model predicts that using an alternative sensory system with less noise would allow the bat to apply stronger turning forces. In the highly visual Egyptian fruit bat [22], visual estimation of *θ* is less noisy than echolocation-based estimates. This is due to two factors: First, visual angular acuity in these bats is 0.3° [22], 10-fold better than their echolocation-based acuity of 2°–3° [17]; and second, vision has a much higher effective update rate (at least 25 Hz flicker-fusion limit in mammalian vision [32] versus 7–10 Hz echolocation rate). We therefore hypothesized that, because the effective sensory noise is substantially reduced under vision, bats performing a visually guided flight will be able to use stronger maneuvering forces *F*_⊥_, which will be expressed by higher gain parameters (*k_p_*, *k_d_* ) in our model.

**Figure 3:**
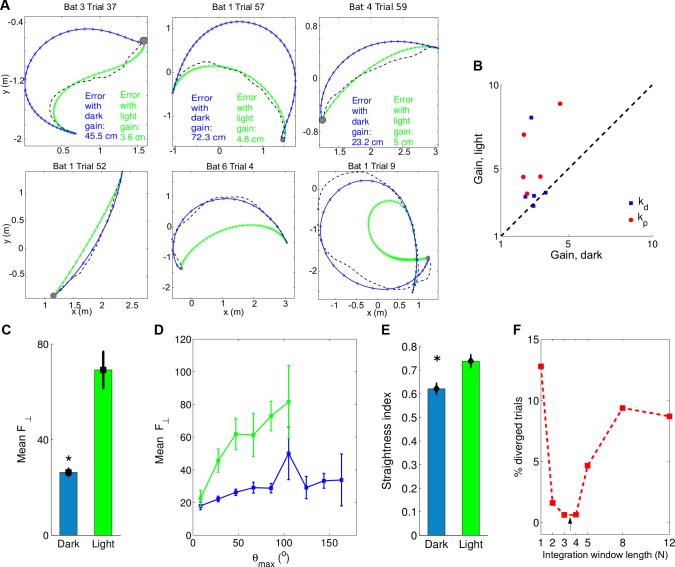
Effects of different sensory inputs (vision versus echolocation) on flight control. (a–c) Six example flights: three recorded in the light (top) and three in the dark (bottom, same examples as in Figs. 1–2). Dashed black curves, real trajectories. Blue curves, trajectory of PD controller using *k_d_*, *k_p_* gains optimized for dark conditions. Green curves, trajectory using *k_d_*, *k_p_* gains optimized for light conditions. Dots represent sensory acquisition time samples (Blue: 10 Hz, echolocation; Green: 25 Hz, vision). The errors between the real and simulated trajectories (indicated inside the figures) show that high gains are preferable in light conditions. (b) Gain parameters of the PD controller in light and dark for each bat (for the five bats that participated in both dark and light experiments). Note that eight of the ten parameters were above the diagonal—indicating larger gain parameters in light versus dark. (c) The forces exerted by the simulated bat (*F*_⊥_) were much stronger in the light than in the dark (see main text). (d) The mean force exerted by the simulated bats increased with the maximum angle *θ*_max_ in both light and dark, but was always stronger for light (green) than for dark conditions (blue). (e) The straightness index of the real bat trajectories (see Materials and Methods) was significantly lower in dark than in light, for initial angles > 45° (*p* < 0.03); this indicates that the bat flies straighter in light conditions. (f) An integration window of length 3 yielded simulations that reconstructed most accurately the real flight trajectories in the light (arrow). All the data is available in the S3 Data.

To test this hypothesis, we conducted new experiments in which the same individual bats (five of the six bats) flew under a light level that is considered optimal for bat vision (1 lux) [33], while performing the same landing task. We examined here all of these 43 light-based trials, and found that the PD controller used for modeling the dark conditions (Figs. 1 and 2) was inadequate in explaining the light trials (the mean error was 2-fold higher, see Materials and Methods). Therefore, we implemented a new version of the PD controller, where we increased the sensory update-rate from 10 Hz (sonar) to 25 Hz (vision [32]) and also decreased the noise level [22] (see Materials and Methods). This PD model was able to reproduce the trajectories flown in the light with a high fidelity (Fig. 3A, top row, green curves). We found that, as we hypothesized, the simulated bat exhibited significantly larger gain parameters (*k_p_*, *k_d_* ) in light versus dark (Fig. 3B; 8/10 parameters increased in the five bats; Wilcoxon signed rank test, *p* < 0.01). Further, the simulated bat exerted significantly stronger forces when flying in the light (Fig. 3C; *t*-test, comparing flights with *θ*_0_ > 45°: *p* < 10^−11^ see also Fig. 3D). Moreover, flight trajectories in light conditions were more direct than in darkness, as quantified by their higher straightness index (Fig. 3E, *t*-test: *p* < 0.03). Additionally, we found that using an appropriate integration window is even more crucial in light than in dark (the optimum is more pronounced, see Fig. 3F, arrow).

We next tested if the light-based gain parameters could explain trajectories flown in the dark, and vice versa. When we applied on flights performed in light the *k_p_*, *k_d_* parameters derived from dark trials, the simulations produced trajectories that converged onto the target, but were much less direct (Fig. 3A, top row, blue curves). Conversely, when we applied on dark trials the *k_p_*, *k_d_* parameters from the light condition, the simulations produced straighter trajectories (Fig. 3A, bottom row, green curves; and Materials and Methods), consistent with the stronger forces applied by the bats in the light. Interestingly, this implies that, in terms of its motor abilities, the bat could have easily flown more directly to the target in the dark—but instead, it flew in a curved manner because of sensory limitations (namely, the higher sensory noise and lower sensory update-rate in the dark).

## Discussion

Taken together, our results demonstrate a simple, biologically-plausible, sensorimotor model that explains flight guidance in bats—that is, how they maneuver towards a desired target in the presence of sensory noise. In our model we concentrate on translating noisy sensory input into motor commands. We ignore noise in the motor and proprioception systems [34], both of which affect the bat’s flight performance in reality. Such noise should be similar in light and in dark and thus will not affect any of our results (aside from making the flight trajectories less smooth).

Our model relies on two key components. The first is (i) integrating (averaging) sensory information over time, in order to acquire a better (less noisy) estimation of the angle-to-target (*θ*). Importantly, we have found that the bat could apply a very simple biologically-plausible implementation of such an estimator (or integrator), which uses minimal memory and only requires memorizing one previous sensory estimation of *θ* (see Materials and Methods). And the model relies on (ii) the derivative of *θ* in addition to *θ* itself, in order to stabilize the controller. These two components suggest strong limitations on the processing that must be implemented in the bat’s brain, or in the brain of other rapidly moving animals.

We found that two main factors explain why bats fly in curved trajectories towards the target: (i) The initial angle *θ*_0_: when the bat starts flying at a non-zero initial angle *θ*_0_ (e.g., Fig. 1C), it flies in a curved manner, while if the bat takes off from the wall with a near-zero *θ*_0_, it flies rather straight to the target (Fig. 1B). This is especially true in the dark (when sensory noise is higher) and the bat does not apply strong forces to decrease its angle-to-target. (ii) The gain parameters *k_p_*, *k_d_* of the PD controller: low gain parameters are used when the sensory noise is higher (e.g., in the dark), leading to curved flights; while high gain parameters lead to straighter flights.

Finally, the experiments in the light suggest that, when using vision, bats can exert stronger motor command (stronger forces) and thus fly to the target more directly than when using biosonar. In contrast, in the dark, when there is higher sensory noise and lower sensory update-rate, the bats exert weaker forces—and therefore the angle-to-target converges more slowly. Taken together, these results imply the surprising conclusion that the highly curved flight trajectories often exhibited by bats in the dark are due to sensory limitations—not motor limitations.

## Materials and Methods

### Experimental Procedures

All experimental procedures were approved by the Institutional Animal Care and Use Committees of the Weizmann Institute of Science and the University of Maryland, where these experiments were performed.

The full behavioral methods for the dark experiments were described elsewhere [17]. In brief: Six adult Egyptian fruit bats (*Rousettus aegyptiacus*) were trained to detect, localize and approach a polystyrene sphere (10 cm diameter) that was mounted on a vertical pole positioned inside a large anechoic flight-room (6.4 × 6.4 × 2.7 m). The target’s size resembles the size of some fruits that are commonly eaten by these bats in nature, such as mango. To exclude the possibility of using vision, the target was painted black and the room was in complete darkness (illuminance < 10^−4^ lux). To prevent use of olfaction, the bats were food-rewarded (with a piece of fruit) only after landing on the target. After every trial, the target was randomly repositioned inside the room, both in the horizontal and in the vertical planes. A total of 253 trials were collected with at least 30 trials per bat. Because our model only addresses azimuth measurements, *θ*, we excluded all flights in which there was a change of more than 15% in the bat’s elevation (height) over the entire flight. This resulted in 222 trials of darkness flights, at least 20 trials per bat, which were analyzed here. To test the effect of a sensory system with lower noise (vision), additional 43 new trials were conducted in light conditions, using 5 of the 6 individual bats that were tested in the dark. We used a relatively dim light (illuminance = 1 lux), which is considered ideal for bat vision [33], and which is equivalent to light levels under bright full moonlight.

#### Video recordings

Two synchronized, high-speed, digital video cameras (Photron, set with a frame rate of 125 frames per second), were used to record the flight of the bats. The direct-linear-transform algorithm was used to measure the three-dimensional location of the bat and other objects (e.g., the target) in the room, using the two camera views, as done previously [17].

### The Model

The model consists of two controllers (one angular controller for turning the bat and one thrust controller for forward movement), and a set of differential equations describing the bat’s motion. We used a fixed sensory update rate of 10Hz for echolocation (i.e., 100 ms steps) or 25Hz for vision (i.e. 40 ms steps). At each sensory update step, the model uses the angle-to-target *θ*, either deterministically or with additive noise (see main text). The noise was added to the value of *θ*, which was based on a single estimation or on an average of several estimations depending on the exact model (main text). The model then uses this *θ* as input for the first controller—the angular controller, which calculates the turning force according to one of the control methods described in the main text and below (proportional controller or proportional-derivative [PD] controller). This turning force is then used by the second controller which calculates the forward thrust. The two forces (turning and thrust) are then integrated in the motion equations to calculate the angle-to-target, *θ*, at the next time step. In between two updates, the simulated bat continues to move with the same forces as used in the most recent calculation.

We calculate the line-of-sight (LOS) distance to target *r*, the velocity vector *ν*, the angle *θ* between *w* and the LOS, and the angular velocity *ω* (see S11 Fig.), by solving the following set of differential equations (the motion equations)

$$\dot{r}(t)=v(t)\cos\theta (t)$$ (1)

$$\dot{v}(t)=-\mathrm{drag}-\text{angular damping force}+\mathrm{thrust}$$ (2)

$$\dot{\theta }(t)=\omega (t)$$ (3)

$$\dot{\omega }(t)=U_{\theta }(t)$$ (4)

where the operator *Ẋ* represents the derivative with respect to time, *d*(*X*)/*dt*. The forces drag, angular damping, thrust, and the turning force *U_θ_*, which is calculated by the angular controller, are explained below. Additionally, we defined *θ_max_* as the maximum angle *θ* along the flight *t* > 0.

The acceleration *ν̇*(*t*) can be described by the following ordinary differential equation (ODE):

$$\dot{v}(t)=-\mathrm{drag}-\text{angular damping force}+\mathrm{thrust}$$

$$=-D_{f}v(t)-D_{t}∥U_{\theta }(t)∥+U_{v}(t)$$ (5)

where drag is proportional to the velocity *ν*(*t*), and *D_f_* is the drag coefficient [11]; and the angular damping force is proportional to the turning force *U_θ_*(*t*) (i.e., when the bat turns, it slows down), with a coefficient *D_t_*.

The forward thrust of the bat *U_ν_*(*t*) is proportional to the wing beat and is described by

$$U_{v}(t)=F\sin(2\pi \beta t)$$ (6)

where *β* is the flapping frequency of the wings, which we set to 10 Hz according to the behavior of the real bats [11], and *F* is the forward thrust of each wing flap, according to the data from [11]. In addition to the azimuth *θ*(*t*), we assume that the bat estimates the distance to the target (for instance, by using bio-sonar). The distance in our model is only used to slow down the approaching velocity when the bat reaches a critical minimum distance to the target. We estimated from our observations this distance to be approximately 0.5 m. Slowing down is modelled by replacing Equation 6 with the damping term *U_ν_*(*t*) = −1.1*ν*(*t*).

In order to compare the simulated bat’s motion to our experimental results, we transformed the bat’s egocentric polar coordinates (*r,θ*) into the Earth Cartesian coordinates *x,y* using the linear transformation *θ_x_ = θ + α*, where *θ_x_* is the angle between the vector of flight and the *x*-axis (see S11 Fig.).

This allows computing the velocity components along *x* and *y* through the following equations:

$$v_{x}(t)=v(t)\cos(\theta_{x}(t)),\;v_{y}(t)=v(t)\sin(\theta_{x}(t))$$ (7)

α(t)=atan2(y(t),x(t)),−π≤α(t)≤π (8)

where the coordinates *x*(*t*) and *x*(*t*) are calculated by integration of *ν_x_* and *ν_y_* at every instance (every 1 ms), given the initial conditions *x*(0), *y*(0), *ν*(0) and *θ*(0), and the function atan2 (*y,x*) is the Matlab (Mathworks) function for arctan(*y/x* ), defined in [*−π,π*]. In the main text we denoted *θ*(0) by the shortened notation *θ*_0_.

Using the linear transformation above, we can then calculate the angular velocity and the angular acceleration as

$$\dot{\theta }(t)=\omega_{x}(t)-\dot{\alpha }(t)$$ (9)

$$\dot{\omega }(t)={\dot{\omega }}_{x}(t)-\ddot{\alpha }(t)$$ (10)

where *α̇*(*t*) and $\ddot{\alpha }(t)$ are the first and second time derivatives of *α*(*t*) (*dα*(*t*)/*dt*) and *d* ^2^ *α*(*t*)/*dt* ^2^, respectively). Combining Eq. 3 and 9 leads to the set of differential equations

$${\dot{\theta }}_{x}(t)=\omega_{x}(t)$$ (11)

$${\dot{\omega }}_{x}(t)=U_{\theta }(t)+\ddot{\alpha }(t)$$ (12)

In order to compute *α̇*(*t*) and $\ddot{\alpha }(t)$, we first define the relative velocity components in Earth coordinates to be

$$V_{TBx}=V_{Tx}-V_{Bx}$$

$$V_{TBy}=V_{Ty}-V_{By}$$

where *V_T_* is the velocity of the target; in our case *V_T_* = 0, since we tested only stationary target. *V_B_* = *ν*(*t*) is the velocity of the bat, and *V_TB_* is the relative velocity (target-bat). The components of the velocity vectors can be calculated using *V_B_x__* = *ν_x_*(*t*) and *V_B_y__* = *ν_y_*(*t*) found in Eq. 7. Then the expression for change in the line of sight, *α̇*(*t*), is obtained by [27]:

$$\dot{\alpha }(t)=\frac{x(t)V_{B_{y}}-y(t)V_{B_{x}}}{x{\left(t\right)}^{2}+y{\left(t\right)}^{2}}$$ (13)

and $\ddot{\alpha }(t)$ is obtained by differentiation of Eq. 13 using the chain rule.

### The Angular Controller

Control theory offers many solutions for how to calculate the amount of forces needed in order to achieve a desired response with respect to performance and stability. In the case of the bat, the objective is to reduce the angle *θ* between the line of flight and the LOS, by applying turning forces *U_θ_*(*t*) (in the main text denoted *F*_⊥_), thus controlling the angular acceleration of *θ*. In our case of a stationary target, the control objective is to reduce *θ* to zero. The simplest control strategy that can achieve this is the so-called proportional control, applying forces that are proportional to the error *e*(*t*); in our case of reducing *θ*(*t*) to zero, the error *e*(*t*) is *θ* itself. The forces are computed by

$$U_{\theta ,p}(t)=-k_{p}\theta$$ (14)

where *p* denotes the proportional controller, and *k_p_* is the proportional factor (gain). This angular force *U_θ,p_*(*t*) is then used in Eq. 4 to calculate $\dot{\omega }(t)$—the change in angular velocity. A typical problem of this proportional controller is that it does not consider the rate of convergence, and therefore tends to overshoot and often oscillates around the desired angle. In certain cases, the proportional controller may even lead to divergence away from the target (instability). To compensate for this and to stabilize the system, it is common to introduce a derivative element, which takes into account the rate in which the angle *θ* changes. In the proportional-derivative (PD) controller, the forces are computed as:

$$U_{\theta ,pd}=-k_{p}\theta -k_{d}\dot{\theta }$$ (15)

where *d* denotes the derivative term, *k_d_* is the derivative gain, and *k_p_* is the proportional gain as before. We assume that the bat estimates the angular velocity, for instance by using a simple first order numerical differentiation, such as first order Euler [26], obtaining

$$\dot{\theta }(t)=\frac{\theta (t)-\theta (t-1)}{dt}$$ (16)

where *θ*(*t* – 1) is the previous measurement of *θ*.

Other simple, well-known, two-gain control strategies that we tested were the proportional-integrative (PI) and the derivative-integrative (DI). The PI controller can be written as

$$U_{\theta ,PI}=-k_{p}\theta -k_{i}\int_{t_{0}}^{t}\theta d\tau$$ (17)

where the *k_i_* is the integral gain. The less commonly used, two-gain parameter DI controller can be written as

$$U_{\theta ,DI}=-k_{d}\dot{\theta }-k_{i}\int_{t_{0}}^{t}\theta d\tau$$ (18)

### Model Training and Testing

To estimate the proportional and derivative gains of the controller (*k_p_* and *k_d_*, respectively) that best fit the bats’ behavior in the dark, we first simulated 31 values in the parameter space of each gain, ranging from 0 to 16. We validated the model by randomly dividing the dark trials of each bat into 80% training trials and 20% testing trials (80% of the 222 trials were randomly selected with equal probability). We first computed the best mean gain parameters for each bat in the dark using the training set and then tested these parameters on trials from the test set (the remaining 20%). This procedure was repeated 100 times. The average error for the training set was 4.6 cm, while for the test set it was 14.6 cm, suggesting that our model could indeed predict accurately the bats’ behavior. All the errors reported in this article are errors on the testing set. The test values for each bat are provided in S1 Table.

To test whether our model, developed for dark conditions, is accurate for light conditions as well, we simulated the trials conducted in light for each bat using the same model, i.e., 10 Hz update rate of echolocation and the same gain parameters that were used in the dark; we then compared the error index. Few of the trials diverged, and of the trials that did converge, the mean error was 2-folds larger than the error we obtained for the dark trials, especially for large angles (maximum error over 60 cm). We therefore went on to test a different model for the light with a higher update rate (25 Hz) and a different noise model with less noise (see below).

Interestingly, when we used the training procedure in order to optimize the control parameters in the light trials (see the *k_p_* and *k_d_* values in S2 Table), and then tested these on the dark trials, most of the simulations diverged. Of those simulations that did converge, the error increased from 2.6 cm in the light to 21.50 ±0.6 cm in the dark, supporting our hypothesis that the bat uses a different control model in the light than in dark conditions.

### Control Parameter Space

We tested the parameter space *k_d_* and *k_p_* by simulating the 222 dark trials and 43 light trials, all without noise, over a grid of combinations of *k_d_* and *k_p_* from 0 to 16. We measured the error index between each simulation and its corresponding real trajectory (see below). The parameter values that minimized the error index are provided in S2 Table.

### Convergence Criteria

A trial simulation was stopped when one of the following convergence criteria was satisfied: (1) When the simulated bat hit the target (i.e., reached a 5 cm distance from the center of the 10 cm diameter target). (2) When the simulation lasted more than 7 s. This criterion was set because the average time-to-landing in the real experiments was 1.5 s, and the longest flight duration observed in the 222 trials was less than 4 s.

### The Error Index

In order to compare the trajectories of the simulated and the real bat, we first created a joint closed shape that is defined by the two curves (i.e., as illustrated in S12 Fig., the curves that surround the areas *A*_1_, *A*_2_, and *A*_3_). The area inside the closed continuous curve can be calculated by

$$∥A∥=\iint_{D}dA=\frac{1}{2}\oint_{C}(xdy-ydx)$$ (19)

Since our vectors are in discrete form, the above continuous equation can be approximated to discrete-time form by the following

$$A_{j}=\frac{1}{2}[\sum_{i=1}^{m-1}(x_{i}+x_{i+1})(y_{i+1}-y_{i})-\sum_{i=1}^{m-1}(y_{i}+y_{i+1})(x_{i+1}-x_{i})]$$ (20)

where *m* is the number of discrete points in each curve. If the curves intersect, the intersection points are found using linear interpolation between each of the elements composing the curves (S12 Fig.). In those cases, we sum all the closed areas using

$$∥A∥=\sum_{j=1}^{k-1}A_{j}$$ (21)

where *k* is the number of intersection points (e.g. in S12 Fig., *k* = 4). The error index is defined by normalizing the total area ∥ *A* ∥ by the length of the experimental trajectory of the corresponding trial. Simulations in which the bat circled the target prior to landing (see convergence criterion above) were penalized by multiplying the error by a factor of 10, since such a behavior was never observed in the real experiments.

### The Noise Model

In the dark, we assumed that the bat measures the angle, based on echolocation with a frequency of 10 Hz, i.e., every 0.1 s. We assumed that the noise is *θ*-dependent [20–22], and we tested two different models. The first noise model has no bias (the measured *θ* linearly depends on the real *θ*), and it has a nonlinear noise term that is low at small angles and increases above *π* / 2 (S9 Fig.—left):

$$\tilde{\theta }=\theta +n\kappa_{1}\sin^{2}\theta +n\kappa_{2}$$ (22)

where $\tilde{\theta }$ is the noisy estimation of *θ*, *κ*_1_ = 0.65, *κ*_2_ = 0.15 and *n* is Gaussian noise (N(0,1)).

The second noise model (S9 Fig. - right) has both a bias in the estimation of *θ*(*t*) and additive Gaussian noise that is *θ*-dependent [20–22]:

$$\tilde{\theta }=a\sin(\theta b)+0.4n\theta +0.15n$$ (23)

with the parameters *a* = 2.22 and *b* = 0.458, and *n* is the Gaussian noise. In the light conditions, the simulated bat estimated the angle-to-target *θ* by using both vision and echolocation (the real bats did not completely stop echolocating at this light level). We assumed that the additional independent visual measurements were acquired at a rate of 25 Hz (see more details in the main text). Additionally, the noise model assumed a measurement of *θ*, which is approximately 7-fold more accurate at each time step, following previous behavioral and anatomical studies (see main text):

$$\tilde{\theta }=a\sin(\theta b)+0.07n\theta +0.02n$$ (24)

### Sensory Integration

We implemented the exponentially decaying integration function as follows:

$$\bar{\theta }=\frac{\sum_{i=1}^{N}W_{i}\cdot {\tilde{\theta }}_{i}}{\sum_{i=1}^{N}W_{i}}$$ (25)

where $\bar{\theta }(t)$ is the estimated angle *θ* at the current time after integration (averaging) over several sensory measurements, and ${\tilde{\theta }}_{i}$ is the measured angle *θ* at sample time *i*, with noise and without integration. Each of the previous N sensory inputs was integrated with a weight *W_i_* calculated according to *W_i_ = w* ^*i*–1^ / *V* and *V* = (1–*w^N^*)/(1–*w*), and *w* is the decay parameter, which was estimated in our case to be *w* = 0.5. The window that was found to best explain the behavior was N = 3–4 (Fig. 2E). We also tested a linearly decaying averaging model, and a uniform averaging model, and found the exponentially decaying average to be superior in terms of successful convergence and accuracy (error index); this testing was done by comparing between the experiments trajectories and 120 simulated trajectories with noisy measurements for each trial (S10 Fig.).

The above integration function requires the memory of at least three past measurements, and to perform a relatively complicated mathematical expression at each time step. A much more practical and biologically plausible method to implement the above integration function is to use the discrete form of a low pass filter [26], which updates the current angle estimation using the previous one according to the weight constant *γ*

$${\bar{\theta }}_{k}={\bar{\theta }}_{k-1}(1-\gamma )+\gamma \cdot {\tilde{\theta }}_{k}$$ (26)

where *k* is the time step and ${\tilde{\theta }}_{k}$ is the current noisy measurement of the angle (Eq. 22–24). Using the above equation, the organism needs only to remember the most recent measurement and the value ${\bar{\theta }}_{k-1}$ estimated at the previous measurement. The above practical implementation is another presentation of Eq. 25. To see that, we apply Eq. 26 recursively:

$${\bar{\theta }}_{k}=\gamma {\tilde{\theta }}_{k}+\left(1-\gamma \right)\gamma {\tilde{\theta }}_{k-1}+{\left(1-\gamma \right)}^{2}\gamma {\tilde{\theta }}_{k-2}+\cdots +{\left(1-\gamma \right)}^{k}{\bar{\theta }}_{0}$$

$$=\sum_{i=0}^{k-1}{\left(1-\gamma \right)}^{i}\gamma {\tilde{\theta }}_{k-i}+{\left(1-\gamma \right)}^{k}{\bar{\theta }}_{0}$$

and for the choice of *γ* = 0.5 and assuming that the first estimation ${\bar{\theta }}_{0}=0$, we have

$${\bar{\theta }}_{k}=0.5{\tilde{\theta }}_{k}+0.25{\tilde{\theta }}_{k-1}+0.125{\tilde{\theta }}_{k-2}+0.0625{\tilde{\theta }}_{k-3}+\cdots$$ (27)

which is corresponding to Eq. 25 with a decay parameter *w* = 0.5. Indeed, the simulation results of the above equation and an exponentially weighted function (Eq. 25) with three past measurements are very similar.

### Proof of Convergence

We have shown (S8 Fig.) that the PD controller was superior in performance to the other two-parameter controllers, i.e. the DI and the PI. It was also superior to the single parameter P controller. We will prove here that the derivative term is essential for stabilization of the guidance controller.

Applying the proportional controller (Eq. 14) to the model dynamics (Eq. 12), we get

$${\dot{\omega }}_{x}={\ddot{\theta }}_{x}=-K_{p}(\theta_{x}-\theta_{d})+\ddot{\alpha }(t)$$ (28)

$$=-K_{p}(\theta_{x}-\alpha )+\ddot{\alpha }(t)$$ (29)

since *θ_d_* = 0. Rearranging the above equation, we get

$$({\ddot{\theta }}_{x}-\ddot{\alpha })+K_{p}(\theta_{x}-\alpha )=0$$ (30)

The solution for this differential equation is marginally stable [24,25] and the solution oscillates; i.e., it never converges to an isolated steady state, except when the true solution is a straight line (initial angle to target is identically zero). Applying a proportional-derivative controller, (Eq. 15) we get

$${\dot{\omega }}_{x}={\ddot{\theta }}_{x}=-K_{p}(\theta_{x}-\alpha )+K_{d}({\dot{\theta }}_{x}-\dot{\alpha })+\ddot{\alpha }$$ (31)

and then we get the second order differential equation

$$({\ddot{\theta }}_{x}-\ddot{\alpha })+K_{d}({\dot{\theta }}_{x}-\dot{\alpha })+K_{p}(\theta_{x}-\alpha )=$$ (32)

$$\ddot{e}(t)+K_{d}\dot{e}(t)+K_{p}e(t)=0$$ (33)

which is an exponentially stable system for any positive parameters *K_p_* ≥ 0 and *K_d_* > 0 [24,25], and thus the error *e*(*t*) = *θ_x_ – α = θ* is reduced to zero exponentially with time. This proves that the derivative term is essential for ensuring convergence of the controller—that is, the PD controller is the simplest stable controller for guidance purposes.

## Supporting Information

S1 Data Individual data points underlying the analyses displayed in Fig. 1. (XLS)

S2 Data Individual data points underlying the analyses displayed in Fig. 2. (XLS)

S3 Data Individual data points underlying the analyses displayed in Fig. 3. (XLS)

S1 Fig Simulations of three trials. Top: Trajectories of the real bats (dashed black), simulated bats with proportional controller (red) and with PD controller (blue curves). Middle panels (red)—depicts proportional controller, the simulated angle *θ* (solid), the real angle *θ* (black dashed-dotted) and the simulated angular velocity *dθ/dt* (dashed). Bottom panels (blue)—the same as in the middle but for the PD controller.
(TIF)

S2 Fig Additional simulation of trials, bat 1. Flight trajectories of the real bat number 1 (black dashed), simulations with optimal dark *k_d_* and *k_p_* (blue), simulations with the same initial conditions but with optimal light gains *k_d_*, *k_p_*, and a pure proportional controller, *k_d_* = 0 (red). The angle *θ*(*t*) (solid), and angular velocity (dashed) are given below the x—y planes, together with the measured *θ*(*t*) (dotted) and the errors between the trajectories for each gain set. The time point of the end of the experiment is marked with grey circle.
(TIF)

S3 Fig Additional simulation of trials, bat 2. Flight trajectories of the real bat number 2; see details in S2 Fig.
(TIF)

S4 Fig Additional simulation of trials, bat 3. Flight trajectories of the real bat number 3; see details in S2 Fig.
(TIF)

S5 Fig Additional simulation of trials, bat 4. Flight trajectories of the real bat number 4; see details in S2 Fig.
(TIF)

S6 Fig Additional simulation of trials, bat 5. Flight trajectories of the real bat number 5; see details in S2 Fig.
(TIF)

S7 Fig Additional simulation of trials, bat 6. Flight trajectories of the real bat number 6; see details in S2 Fig.
(TIF)

S8 Fig Different control strategies. Comparison of the single-parameter Proportional (P) controller (red) to the two-parameters Proportional-Derivative (PD), Derivative-Integrative (DI), and Proportional-Integrative (PI) controllers. Increasing the number of control parameters from one (P controller) to two (PD, DI, PI) does not necessarily decrease the rate of divergence (right) or the error (left) of the trials that did converge. This indicate that the improvement of the PD over the P controller is not due to the number of parameters. Numbers above bars indicate number of diverged trials.
(TIF)

S9 Fig The noise model. Noise envelope in *θ* of the first noise model (Eq. 22, left) and the second model (Eq. 23, right).
(TIF)

S10 Fig Comparison of the three filter types tested. Exponential decaying, linearly decaying and uniform average window, all with window length N = 5; each experiment was compared to 120 simulations with noisy measurements and noise suppression.
(TIF)

S11 Fig Schematic drawing. The bat, the target, and the angle *θ* to the line-of-sight (LOS).
(TIF)

S12 Fig The two trajectories (simulated and real) intersect at four points (marked as +), creating three non-intersecting areas, A1 to A3, that can be calculated using Eq. 20. (TIF)

S1 Table The mean error index (cm) of the six bats, with the S.E.M, for training (a random set of 80% of the dark trials) and testing (the remaining 20% of the dark trials). (TIF)

S2 Table The parameters *k_d_* and *k_p_* that minimize the error index between the simulated trajectories and the real trajectory for each trial in both dark and light conditions. Values are average of *n* trials of each bat, with their respective S.E.M values.
(TIF)

## Abbreviations

θ: azimuth-to-target angle
DI: derivative-integrative
LOS: line of sight
PD: proportional-derivative
PI: proportional-integrative
