# Supplementary figures and images for: A Sensory-Motor Control Model of Animal Flight Explains Why Bats Fly Differently in Light Versus Dark
Source: PLoS Biol. 2015 Jan 28;13(1):e1002046. doi: 10.1371/journal.pbio.1002046 (PMC4309566; doi:10.1371/journal.pbio.1002046)

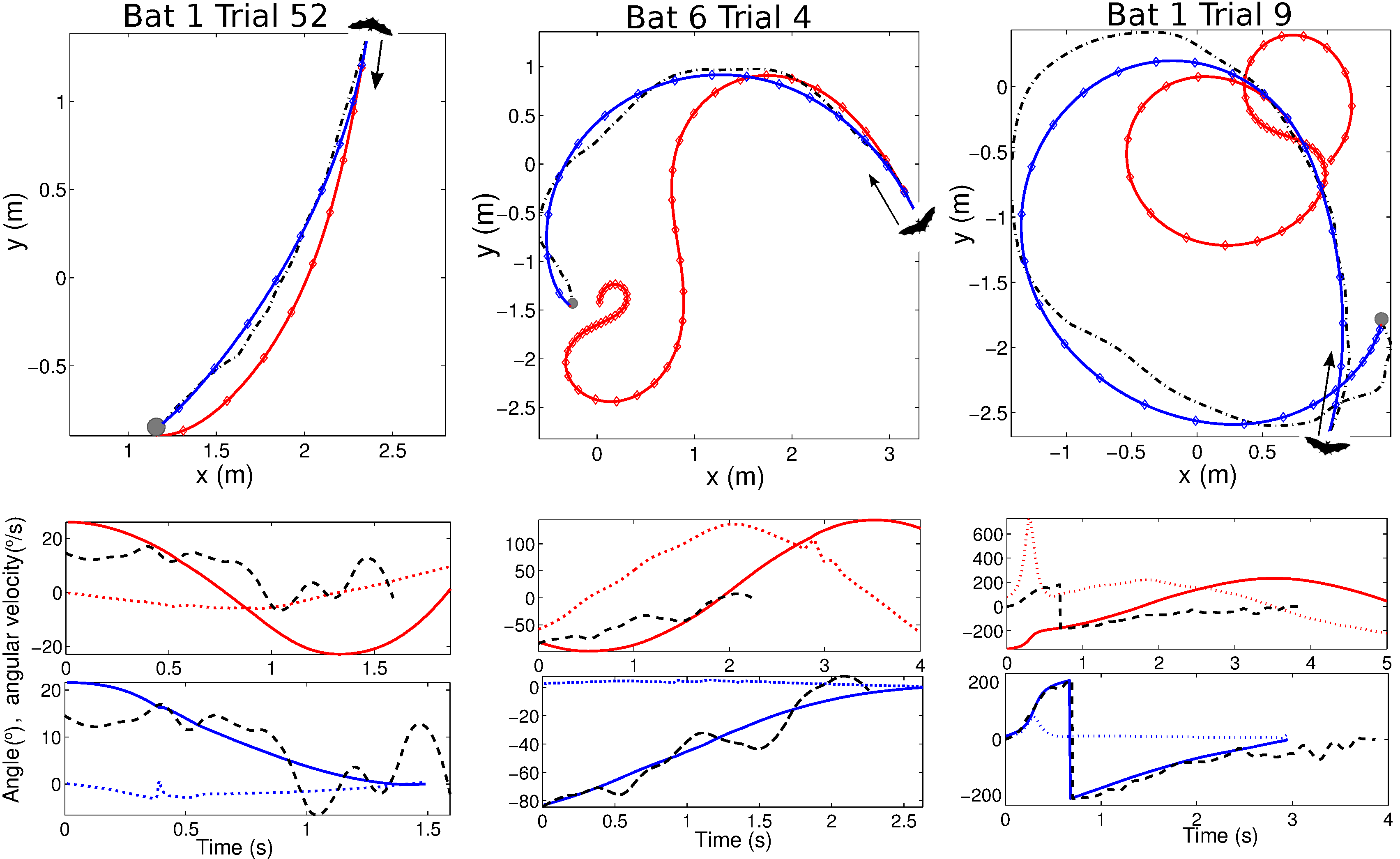

Supplement: S1 Fig — Top: Trajectories of the real bats (dashed black), simulated bats with proportional controller (red) and with PD controller (blue curves). Middle panels (red)—depicts proportional controller, the simulated angle θ (solid), the real angle θ (black dashed-dotted) and the simulated angular velocity dθ/dt (dashed). Bottom panels (blue)—the same as in the middle but for the PD controller. (TIF) [file pbio.1002046.s004.tif]

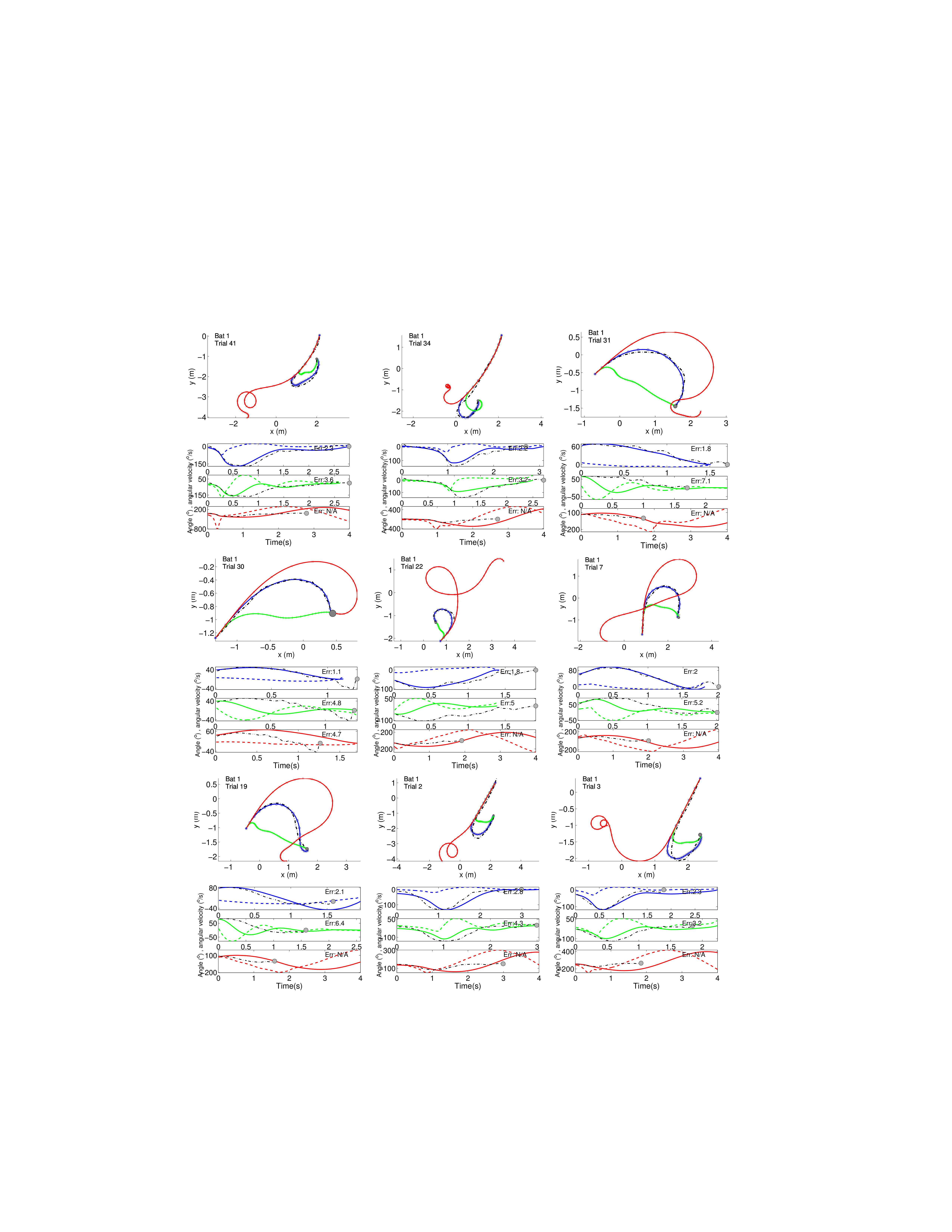

Supplement: S2 Fig — Flight trajectories of the real bat number 1 (black dashed), simulations with optimal dark kd and kp (blue), simulations with the same initial conditions but with optimal light gains kd, kp, and a pure proportional controller, kd = 0 (red). The angle θ(t) (solid), and angular velocity (dashed) are given below the x—y planes, together with the measured θ(t) (dotted) and the errors between the trajectories for each gain set. The time point of the end of the experiment is marked with grey circle. (TIF) [file pbio.1002046.s005.tif]

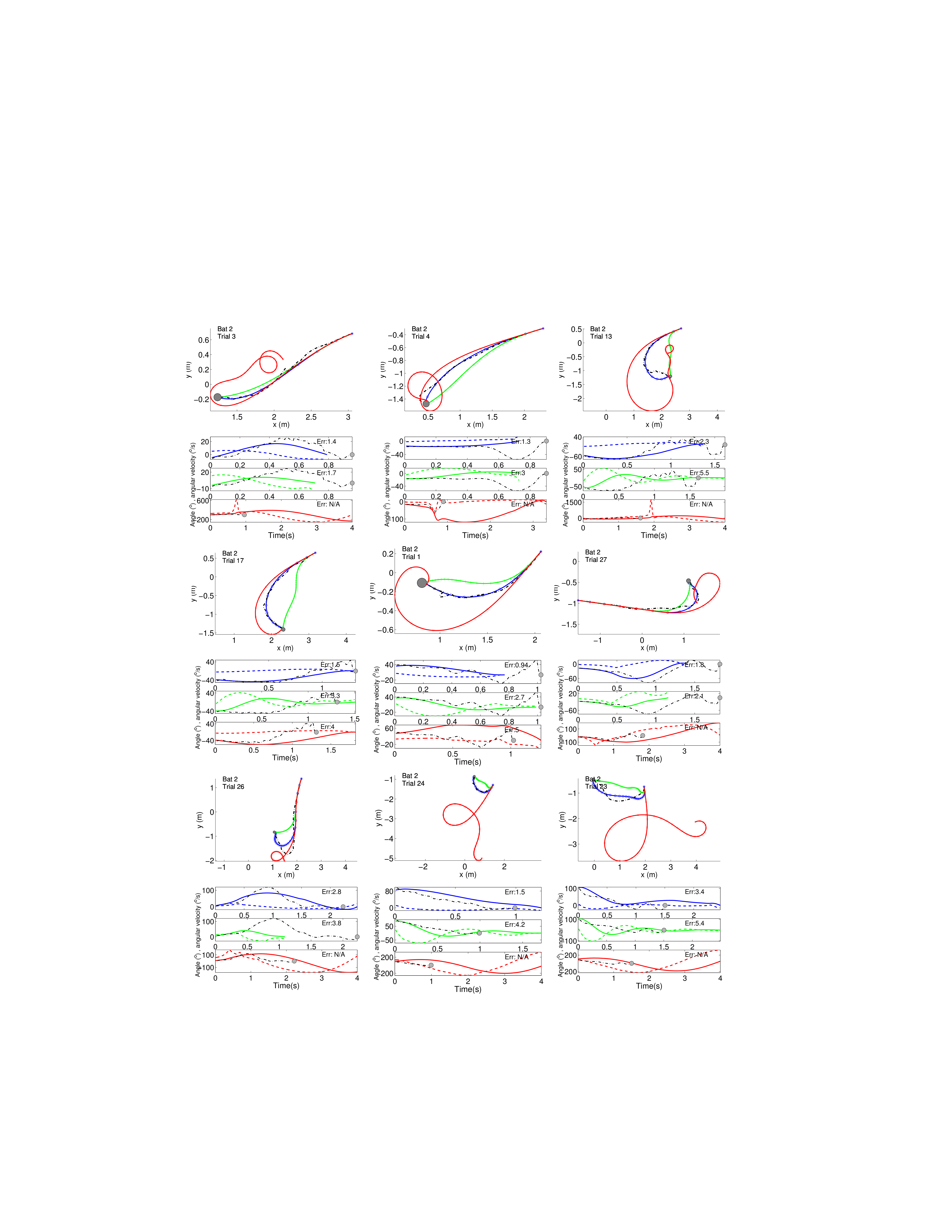

Supplement: S3 Fig — Flight trajectories of the real bat number 2; see details in S2 Fig. (TIF) [file pbio.1002046.s006.tif]

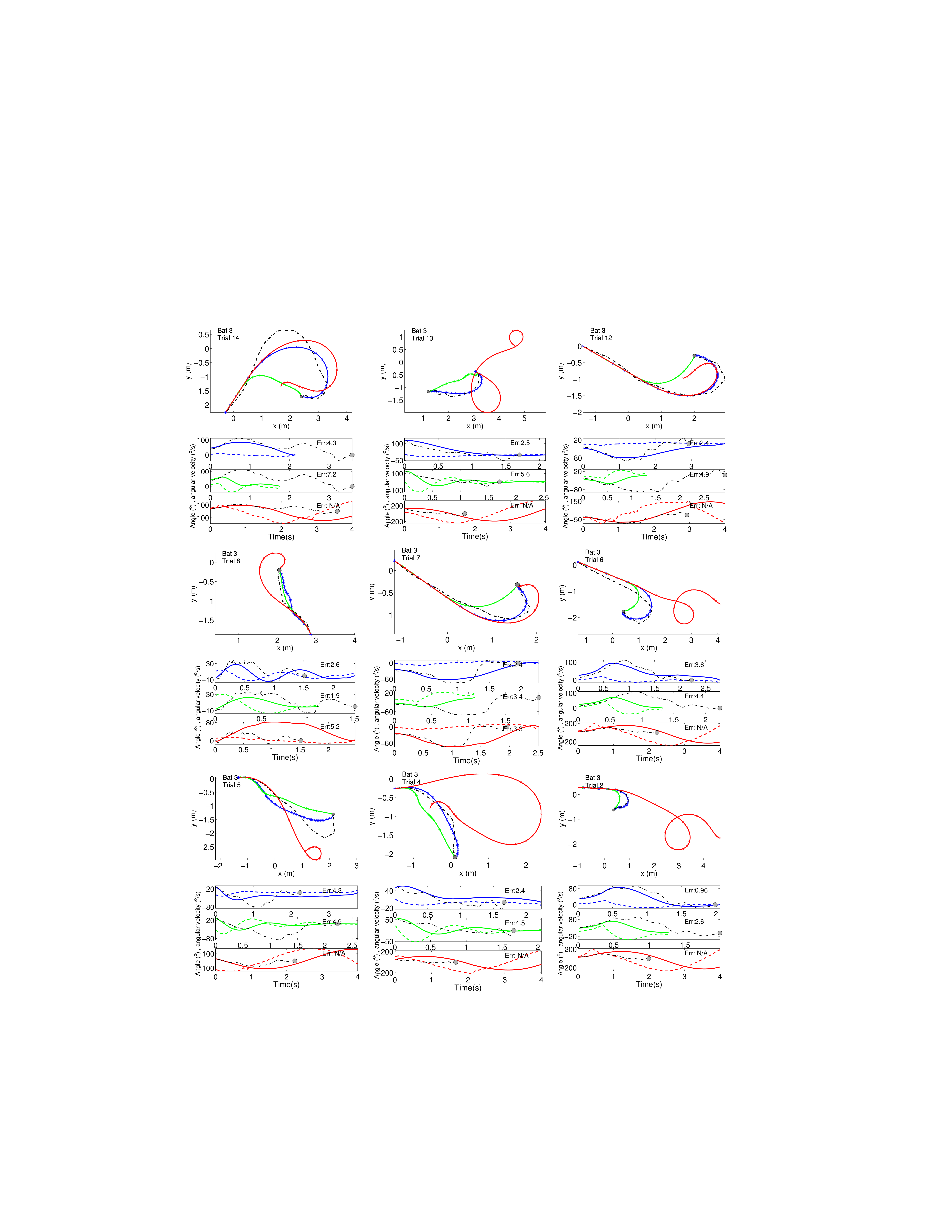

Supplement: S4 Fig — Flight trajectories of the real bat number 3; see details in S2 Fig. (TIF) [file pbio.1002046.s007.tif]

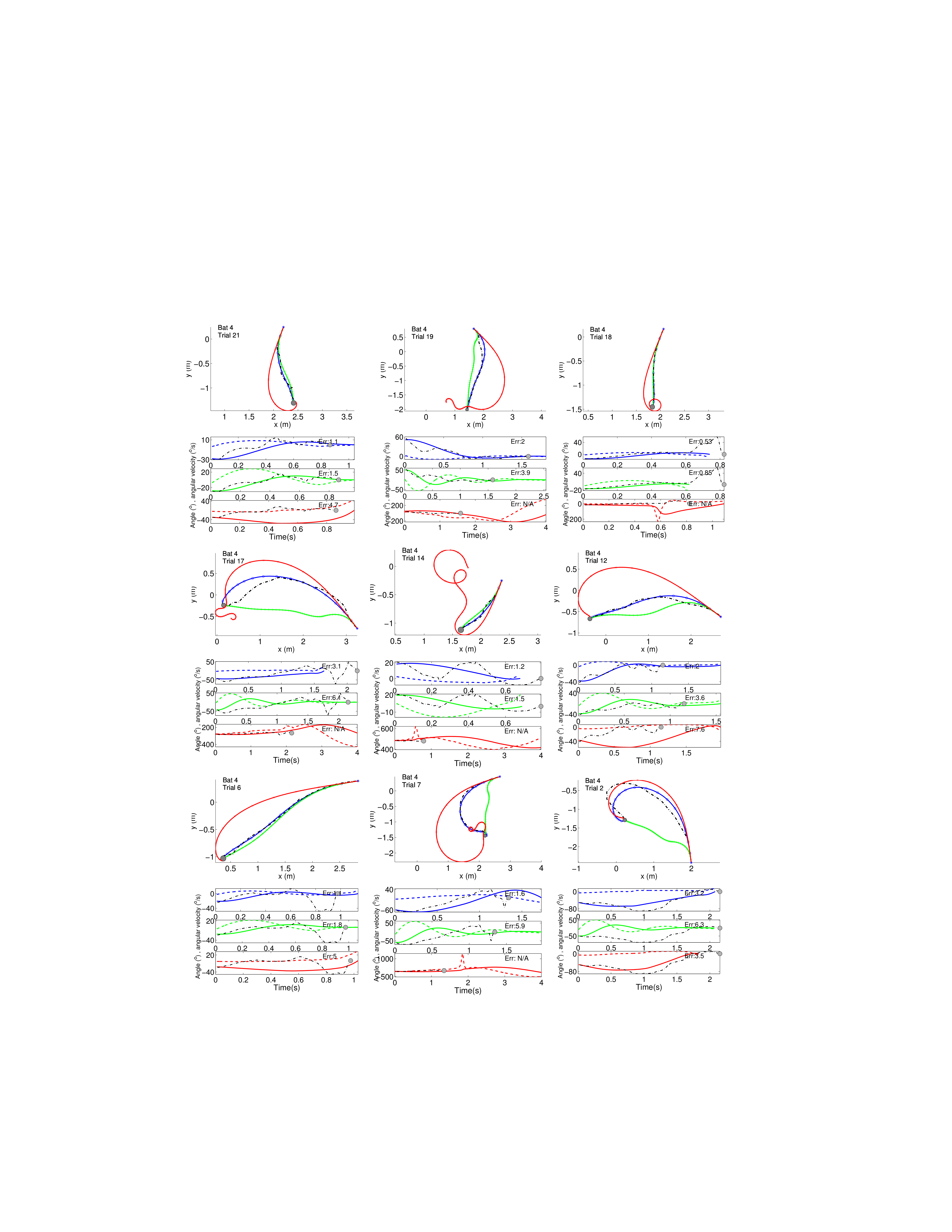

Supplement: S5 Fig — Flight trajectories of the real bat number 4; see details in S2 Fig. (TIF) [file pbio.1002046.s008.tif]

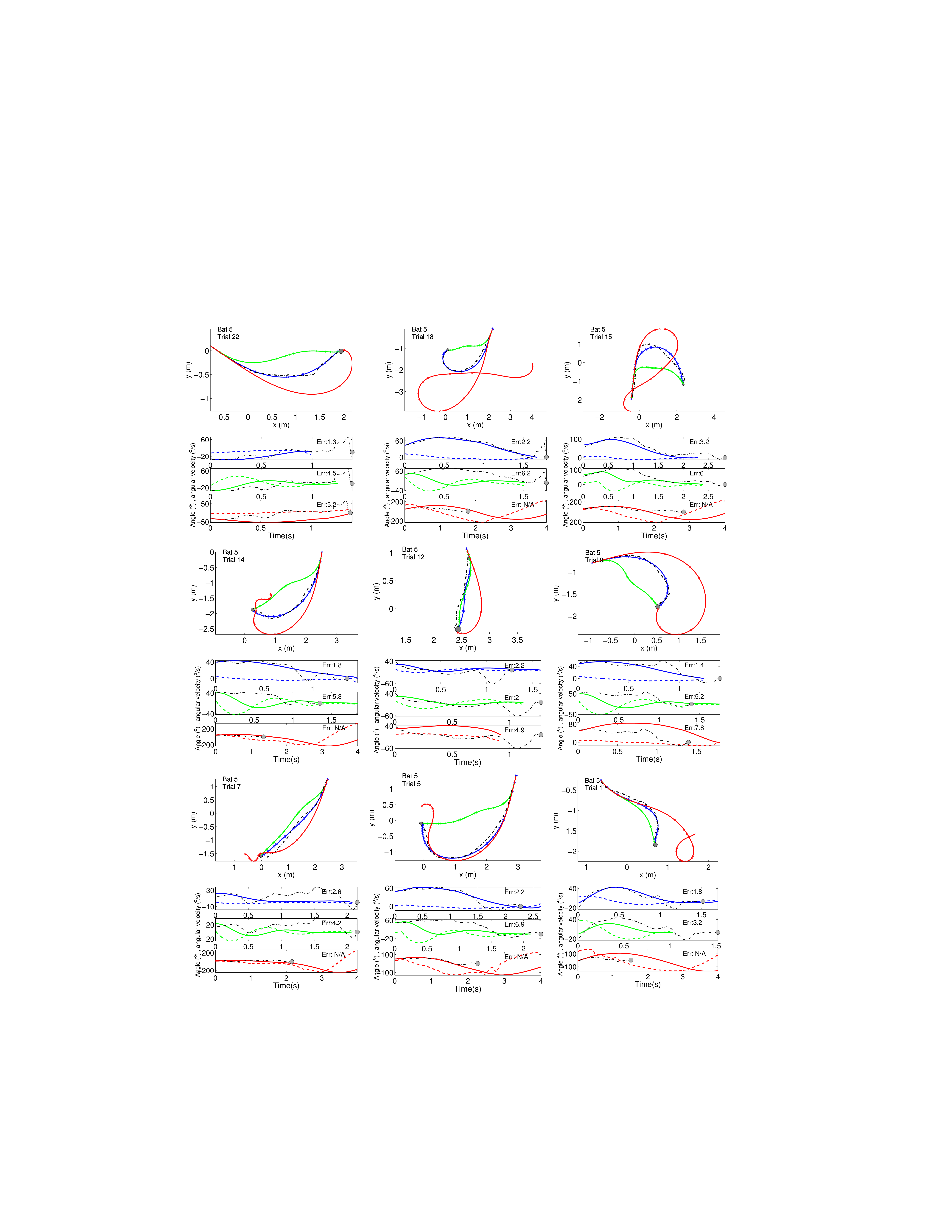

Supplement: S6 Fig — Flight trajectories of the real bat number 5; see details in S2 Fig. (TIF) [file pbio.1002046.s009.tif]

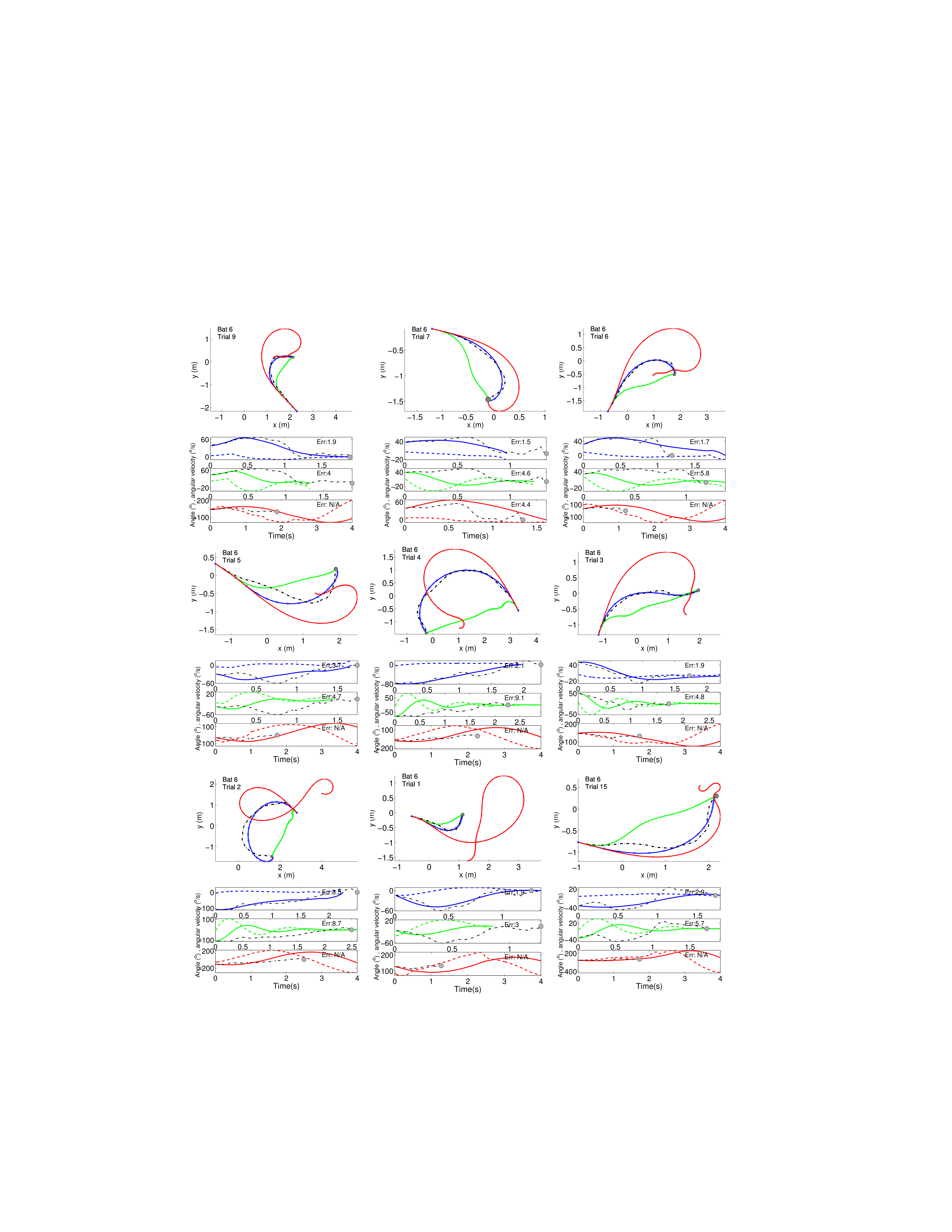

Supplement: S7 Fig — Flight trajectories of the real bat number 6; see details in S2 Fig. (TIF) [file pbio.1002046.s010.tif]

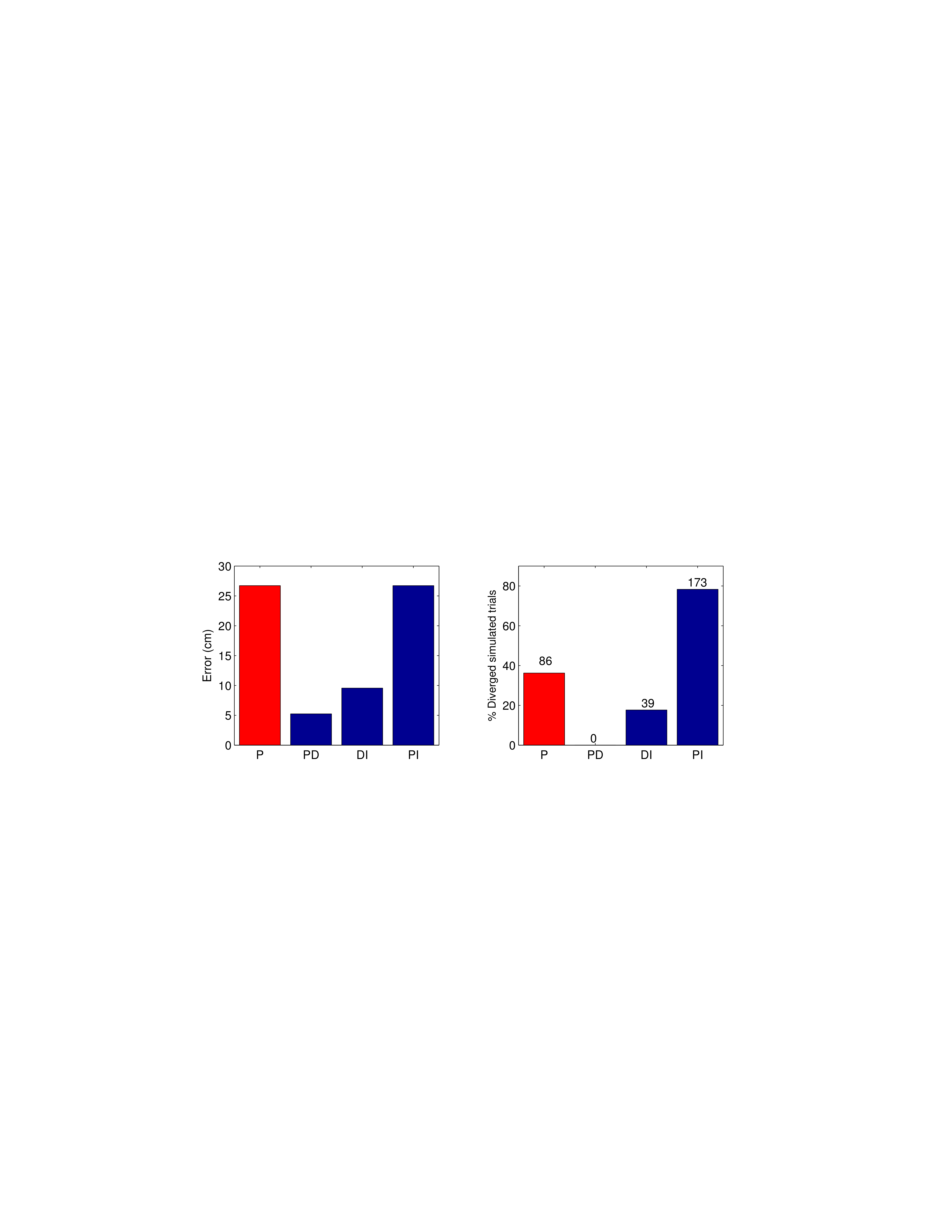

Supplement: S8 Fig — Comparison of the single-parameter Proportional (P) controller (red) to the two-parameters Proportional-Derivative (PD), Derivative-Integrative (DI), and Proportional-Integrative (PI) controllers. Increasing the number of control parameters from one (P controller) to two (PD, DI, PI) does not necessarily decrease the rate of divergence (right) or the error (left) of the trials that did converge. This indicate that the improvement of the PD over the P controller is not due to the number of parameters. Numbers above bars indicate number of diverged trials. (TIF) [file pbio.1002046.s011.tif]

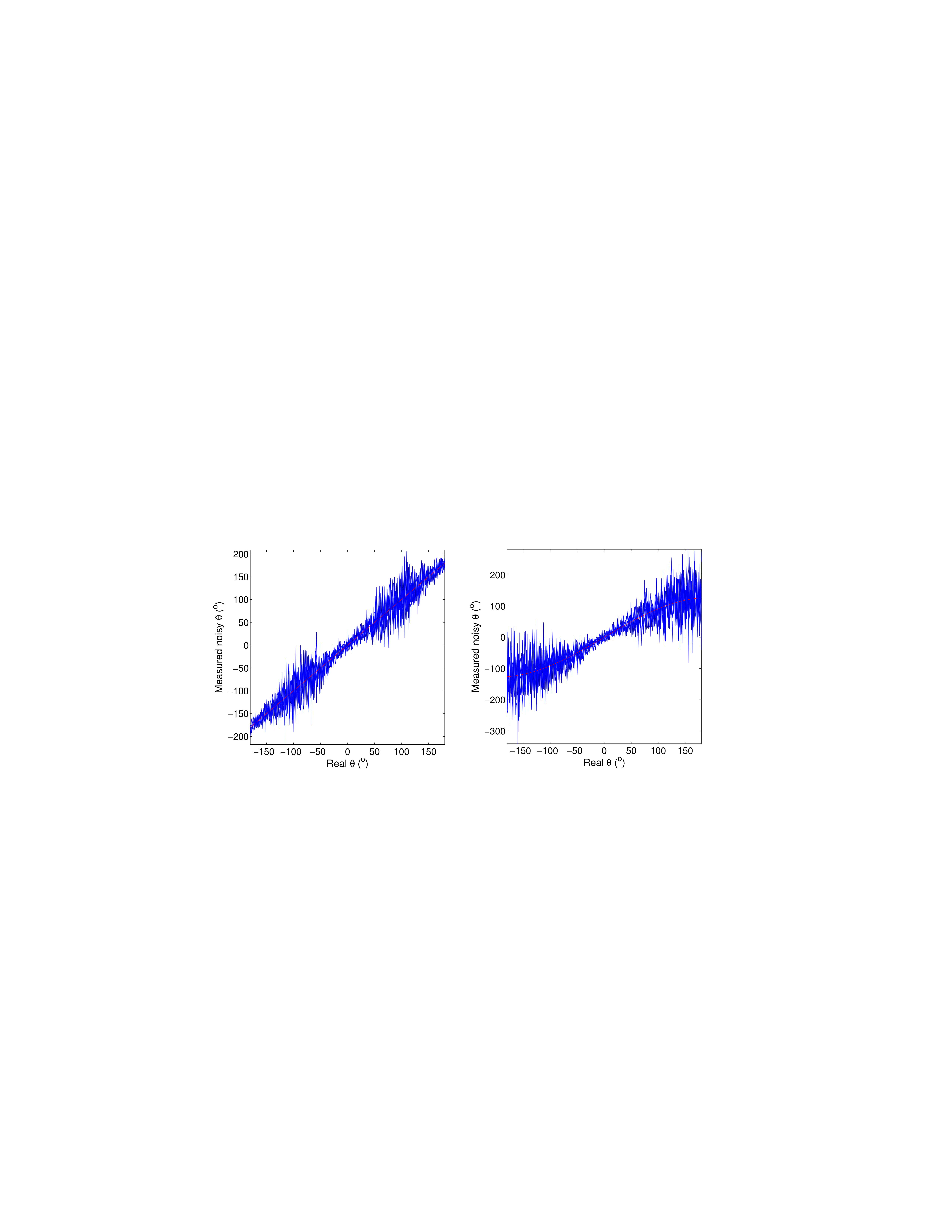

Supplement: S9 Fig — Noise envelope in θ of the first noise model (Eq. 22, left) and the second model (Eq. 23, right). (TIF) [file pbio.1002046.s012.tif]

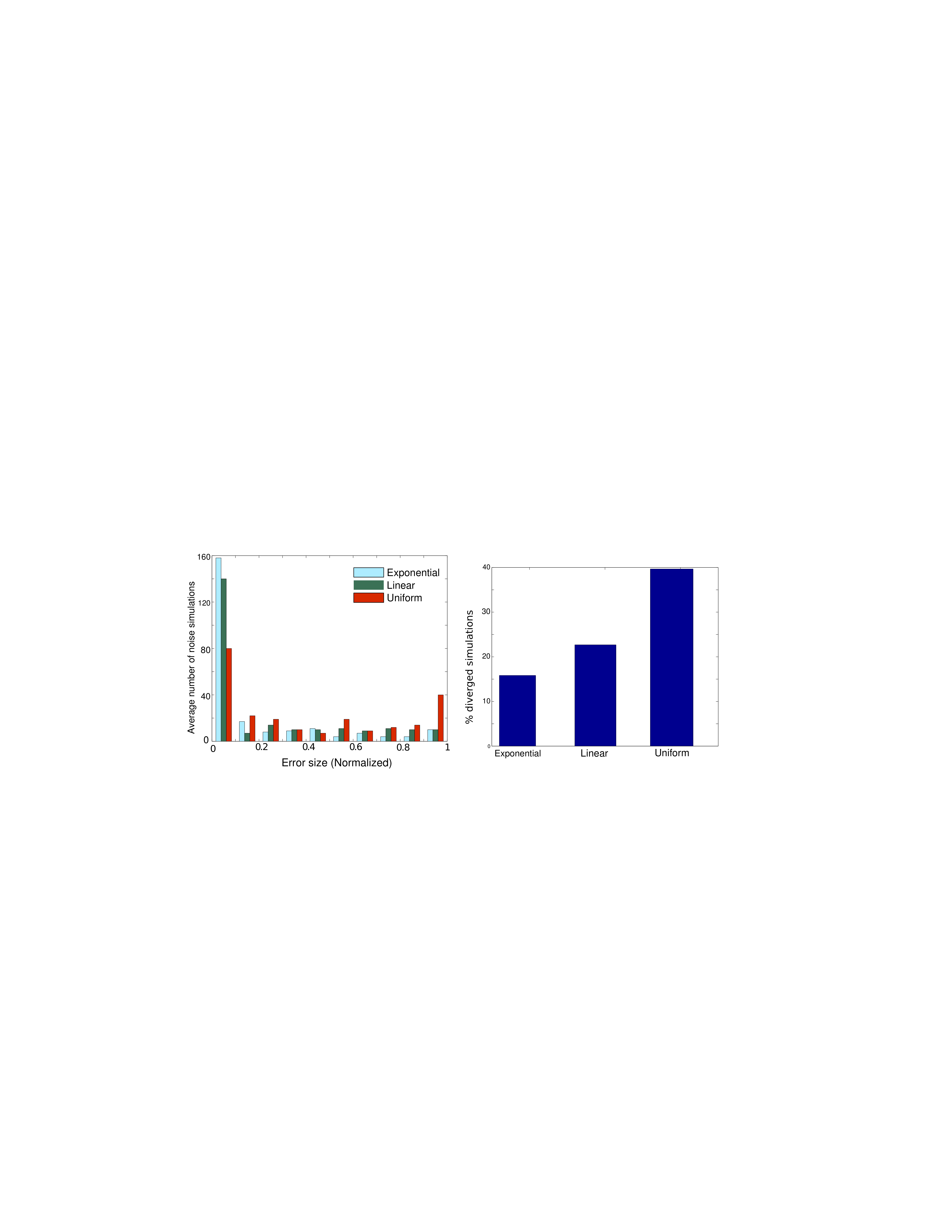

Supplement: S10 Fig — Exponential decaying, linearly decaying and uniform average window, all with window length N = 5; each experiment was compared to 120 simulations with noisy measurements and noise suppression. (TIF) [file pbio.1002046.s013.tif]

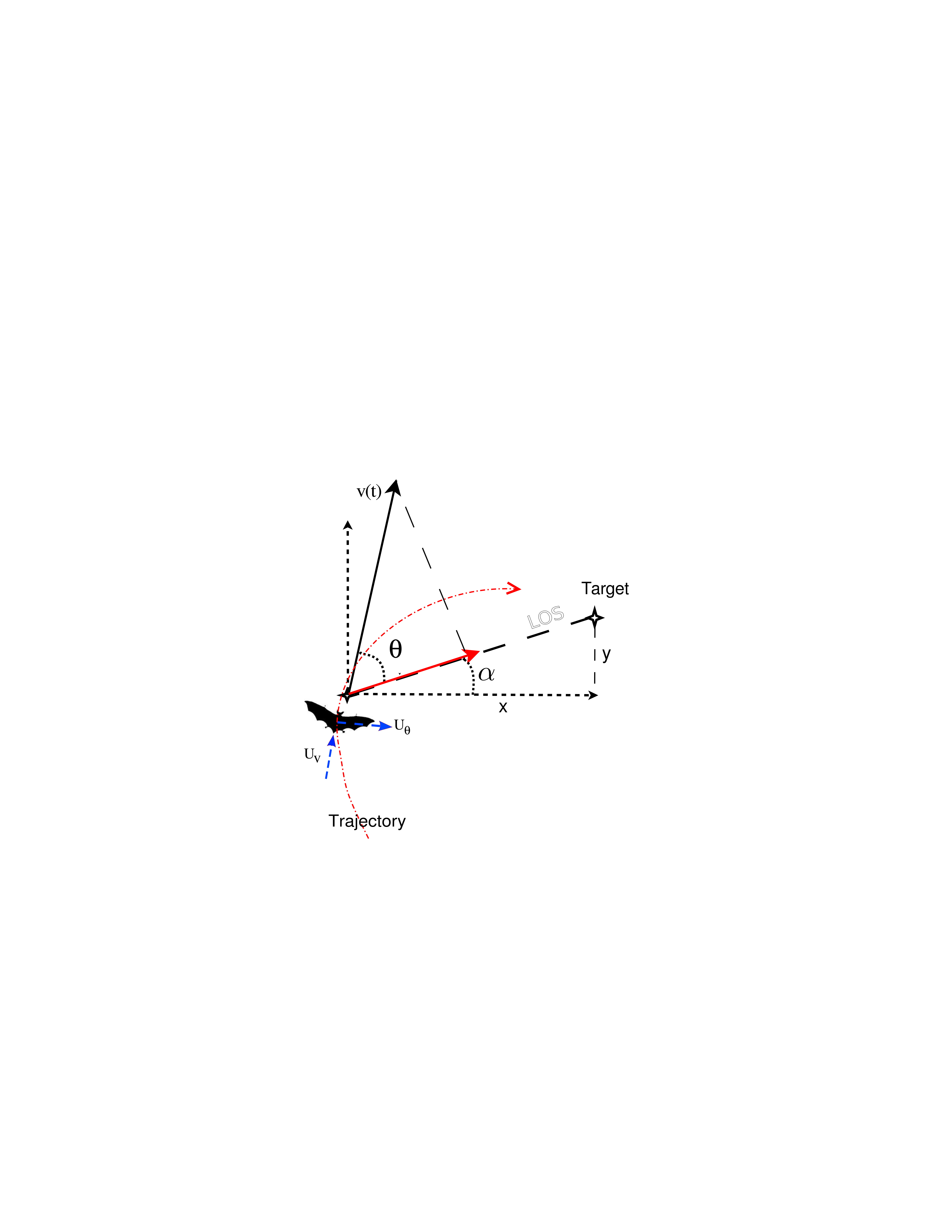

Supplement: S11 Fig — The bat, the target, and the angle θ to the line-of-sight (LOS). (TIF) [file pbio.1002046.s014.tif]

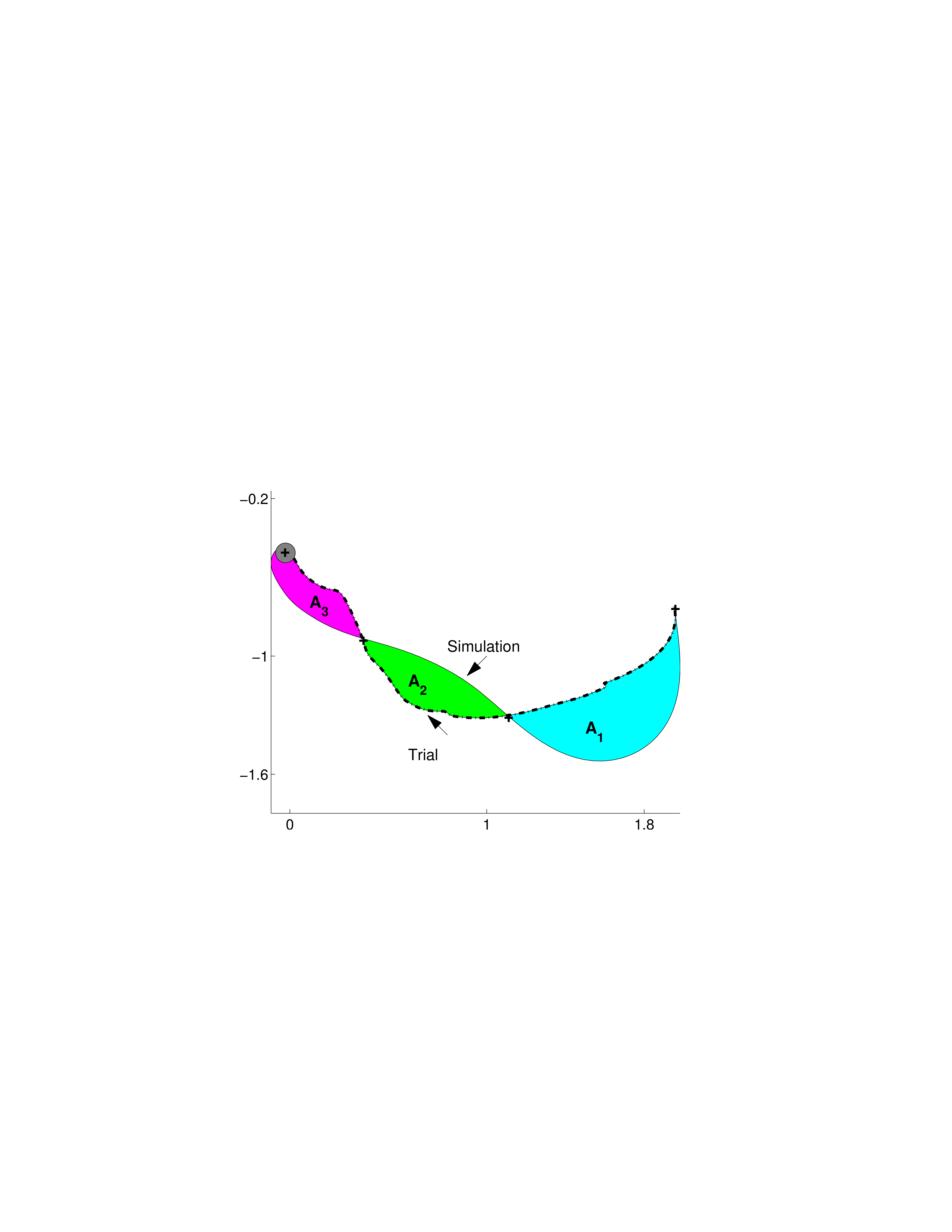

Supplement: S12 Fig — (TIF) [file pbio.1002046.s015.tif]

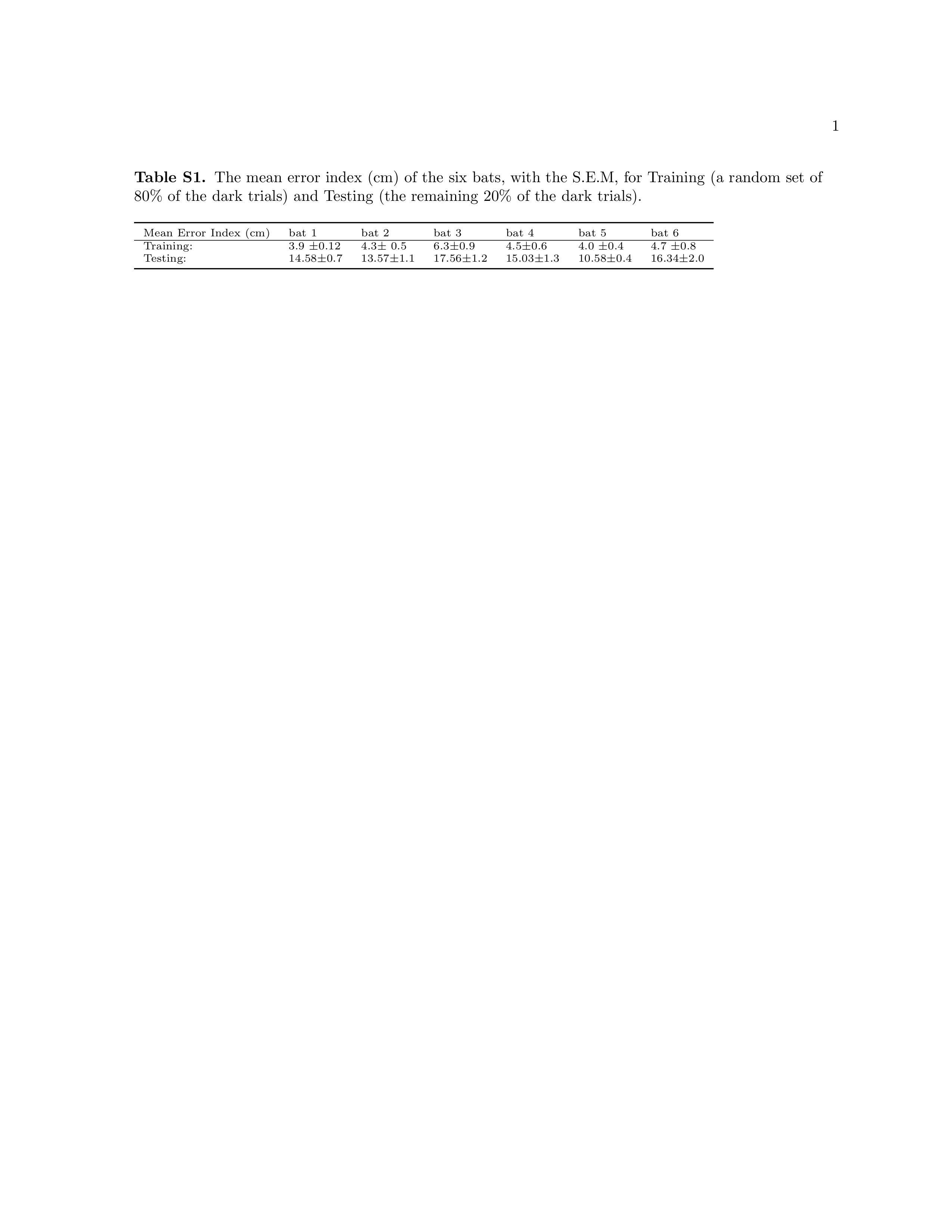

Supplement: S1 Table — (TIF) [file pbio.1002046.s016.tif]

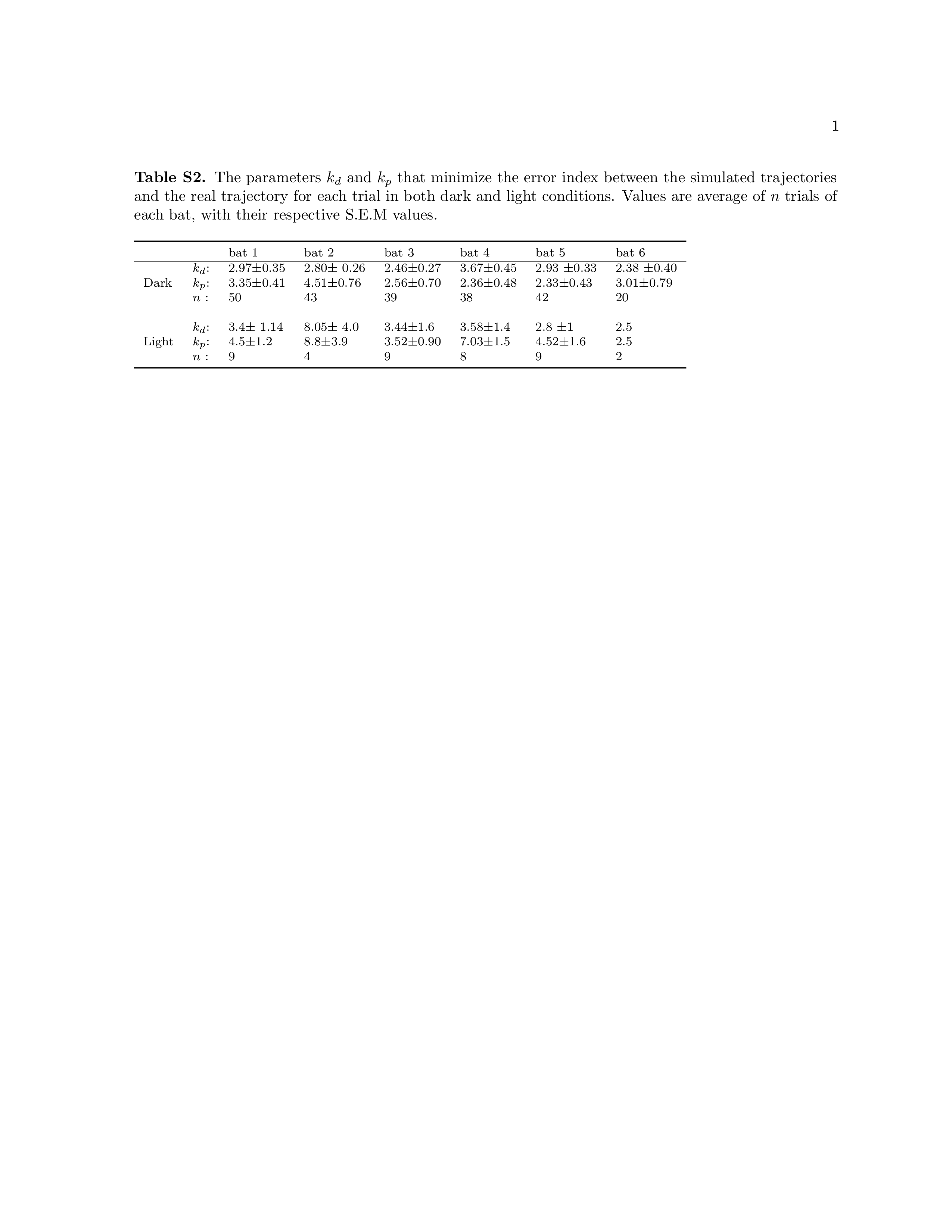

Supplement: S2 Table — Values are average of n trials of each bat, with their respective S.E.M values. (TIF) [file pbio.1002046.s017.tif]
